# Patients’ Perspectives on Applications of AI and Personalized Medicine in Life-Threatening Heart Disease: European Cross-Sectional Patient Questionnaire Study

**DOI:** 10.2196/96968

**Published:** 2026-08-25

**Authors:** Georg Ludwig Lindinger, Nicolas J Schiermeier, Dick L Willems, Menno Tom Maris, Mona Khattab, Hanno L Tan, Dennis Henzler, Marieke A R Bak, Eckhard Nagel, Michael Lauerer

**Affiliations:** 1 Institute for Medical Management and Health Sciences, Faculty of Law, Business & Economics, University of Bayreuth, Prieserstraße 2, Bayreuth, Bavaria, 95444, Germany, 49 921 55 4801; 2 Department of Ethics, Law and Humanities, Amsterdam UMC Location University of Amsterdam, Amsterdam, North Holland, The Netherlands; 3 Amsterdam Public Health Institute, Amsterdam, North Holland, The Netherlands; 4 Department of Clinical and Experimental Cardiology, Amsterdam UMC, University of Amsterdam, Amsterdam, North Holland, The Netherlands; 5 Medical University Lausitz – Carl Thiem, Cottbus, Brandenburg, Germany; 6 Institute of History and Ethics in Medicine, TUM School of Medicine, Technical University of Munich, Munich, Bavaria, Germany

**Keywords:** sudden cardiac death, implantable cardioverter defibrillator, ethics, artificial intelligence, clinical decision support systems, patient acceptance, patient preference, surveys and questionnaires

## Abstract

**Background:**

The growing integration of personalized risk prediction (PRP) and AI substantially reshapes diagnostic and therapeutic decision-making in health care. At the same time, its responsible adoption depends not only on technical performance, but also on patients’ perspectives and acceptance.

**Objective:**

This study systematically examined patients’ perspectives across several European countries and explored how patients’ technology-related attitudes relate to their evaluations of personalized and AI-supported approaches in cardiac care. As part of the PROFID (Prevention of Sudden Cardiac Death After Myocardial Infarction by Defibrillator Implantation) project, its focus is on the ethical use of PRP and AI in the clinical context of decision-making regarding sudden cardiac death (SCD) prevention and implantable cardioverter-defibrillator (ICD) implantation.

**Methods:**

The study used a cross-sectional survey design with a standardized questionnaire including multimedia content. The target population comprised adults aged 18 years or older living in 6 European countries who met at least one of the following (self-reported) clinical criteria: heart failure, myocardial infarction (MI), cardiac arrest, or current ICD implantation. An exploratory factor analysis (EFA) was used to identify and evaluate internally consistent factors, and subsequent regression analyses examined associations between these factors and technological openness, sociodemographic characteristics, and patients’ views on PRP and AI in cardiac care.

**Results:**

The sample consisted of 470 participants from Germany (n=210), the Netherlands (n=86), the United Kingdom (n=145), and 3 other European countries (n=29; Austria, Belgium, and Spain). Overall, 51.9% (244/470) of respondents were male and 48.1% (226/470) were female. The mean age of the sample was 61.12 (SD 12.62) years. The EFA showed six clearly interpretable factors: (1) perceived benefits and support of PRP models in medical decision-making (MDM), (2) perceived benefits and support of AI in MDM, (3) transparency expectations in algorithmic decision-making, (4) support for delegating decisions to algorithms, (5) self-reported AI literacy, and (6) preference for shared decision-making (SDM). The regression analysis showed the relations of technological readiness, self-reported AI literacy, support for delegation of decisions to algorithms, transparency expectations in algorithmic decision-making, preferences for SDM, educational attainment, gender, and age to find associations with patients’ perceived benefits and support of PRP or AI in MDM.

**Conclusions:**

The findings support existing assumptions while also highlighting additional aspects that should be considered if high-level technologies are used in decision-making processes related to ICD implantation. PRP and AI were generally perceived as useful tools to support decision-making regarding ICD indication, provided transparency is ensured and patients remain actively involved in the decision-making process. Mandatory use and full delegation to decision-making directly by AI were broadly rejected. The attributed acceptance of delegation to PRP models was significantly higher than AI. In summary, implementation should support empathetic communication, patient involvement, and individual and institutional responsibility.

## Introduction

### Background

AI is expected to be an important driver of innovation in medicine by advancing diagnosis, treatment, and the efficiency of health care delivery in response to growing demand [1,2]. Although AI has already shown considerable progress in areas such as medical image analysis and natural language processing for electronic health records, its broader integration into routine patient care remains at an early stage [2,3]. Against this background, personalized risk prediction (PRP) models have attracted increasing attention. Their aim is to capture individual risk profiles by integrating a wide range of clinical, imaging, and contextual data, thereby moving beyond rigid threshold-based approaches [4,5]. In this context, PRP models, AI-based systems, and combinations of PRP models and AI application or AI-based PRP models offer new opportunities in decision-making in cardiac care.

Successful implementation, however, depends not only on technical performance but also on how such technologies are perceived by those affected [6,7]. The distinction between these 2 concepts nevertheless is crucial to understanding their perception, even though in reality they are not opposite technologies. To date, research on perceptions of AI in medicine has focused predominantly on health care professionals, who generally report attitudes and expectations regarding its potential to support clinical work [8-13]. Moreover, at least some studies have examined the views of patients with direct or indirect exposure to AI-enabled health care [7,14-18]. Even though the literature explicitly emphasizes the importance of involving patients at an early stage in AI-related projects, this has not yet been adequately achieved in many cases [19,20]. While there are some qualitative studies that elaborated on high-risk applications such as decision-making surrounding implantable cardioverter defibrillator (ICD) implantation in cardiac care [21,22], survey studies examining patients’ perceptions and views remain a research desideratum.

The ethical development and implementation of such technologies require patient-centered research that systematically takes into account expectations, concerns, and limitations from the patient perspective [15,21]. This is particularly important for AI-based clinical decision support tools, which are often not directly visible to patients and therefore cannot easily be individually refused or bypassed [23]. In this context, ethical questions arise regarding perceived benefits, expected transparency, delegating decisions to algorithms, and shared decision-making (SDM).

Overall, the study used a descriptive ethics approach, which aims to understand how normativity exists within a particular context and is based on firsthand empirical research [24,25]. In this regard, this study—entitled PROFID Patient Survey Study—underlines the need for ethically informed ethics studies about these issues, also among patients [24,26]. This study is part of the ethics work package of the PROFID (Prevention of Sudden Cardiac Death After Myocardial Infarction by Defibrillator Implantation) Project (Textbox 1). This includes ethical considerations regarding the implementation of an AI-driven prediction model for sudden cardiac death (SCD) prevention as a life-and-death decision.

Textbox 1. **The PROFID EHRA Trial.**

**PROFID Project [27] – PROFID EHRA Trial [28]**

- **Background**

Sudden cardiac death (SCD) is a major cause of mortality, accounting for around 20% of all deaths in high-income countries [29-31]. In many cases, SCD occurs as a consequence of myocardial infarction (MI). For patients at increased risk, implantation of an implantable cardioverter defibrillator (ICD) represents an effective preventive intervention, including in primary prevention settings, that is, patients who have not suffered an aborted SCD episode [31]. Current European guidelines for primary prevention ICD implantation after myocardial infarction are largely based on severely reduced left ventricular ejection fraction (LVEF <35%) as the main risk criterion [32]. Although reduced LVEF is an established marker of both all-cause mortality and SCD risk, this approach has important limitations: only a minority of patients receiving an ICD experience an appropriate shock, while many SCD events occur in patients who do not meet current eligibility criteria [31]. As a result, there is a substantial mismatch between those who receive an ICD and those most likely to benefit from it. Given that ICD implantation also carries procedural and long-term risks, improving risk stratification remains a key clinical and research priority.

- **Aim of the Project**

The PROFID (Prevention of Sudden Cardiac Death After Myocardial Infarction by Defibrillator Implantation) project [27] aims to reassess routine prophylactic ICD implantation in patients with reduced LVEF (≤35%) after MI in the era of contemporary optimal medical therapy (OMT), with the broader objective of improving clinical practice and reducing the burden of SCD at both individual and societal levels [28,33].

- **The PROFID EHRA Trial**

This noninferiority randomized clinical trial will compare OMT alone to ICD implantation plus OMT in 3595 randomized post-MI patients with symptomatic heart failure and reduced LVEF (≤35%), who would not be eligible to receive an ICD according to current clinical guidelines [28,33].

- **Ethical, Legal, and Societal Issues**

The empirically informed ethics studies address the ethical, regulatory, and governance implications of introducing a personalized approach to ICD implantation, including the use of AI and machine learning. It focuses on issues such as data governance and privacy, patient and professional autonomy, distributive justice, and the role of stratification-based tools in ICD allocation decisions, while also considering emerging challenges throughout the project. The overall goal is to develop an ethically sound framework for personalized ICD decision-making.

### Objective

This study systematically examined patients’ perspectives across several European countries and explored how patients’ technological views and attitudes on technology relate to their evaluations of personalized and AI-supported approaches in cardiac care. The aim was to understand how data-driven decision support is perceived by those affected and which factors shape its acceptance.

The findings of this study were derived from a multinational survey conducted in 6 different countries (Austria, Belgium, Germany, the Netherlands, the United Kingdom, and Spain)—participating in the PROFID EHRA Trial—on perspectives regarding the ethical use of personalized risk prediction and AI in the clinical context of SCD prevention and ICD implantation. In the “Discussion” section, the critical importance of integrating patients’ perspectives into the development and implementation of AI in medicine is highlighted. Although this research is situated in the specific context of SCD prevention, it contributes to broader medical, ethical, and social debates in relation to the implementation of PRP and AI in health-related contexts.

## Methods

### Study Design

The survey uses a cross-sectional survey design using a standardized questionnaire. It is partly based on a semistructured interview study about patients’ perspectives on the ethical use of AI to prevent SCD [21] and a systematic literature review on the perspectives of health care professionals on AI in patient care [9]. Both were conducted by most of the authors and part of the PROFID empirically informed ethics studies.

### Survey Construction and Structure

The questionnaire was developed in British English, consisting of 4 main parts and an additional section about demographic information. To enhance participants’ understanding of key concepts, media content in the form of explanatory graphics and 3 explanatory videos was integrated (Multimedia Appendix 1).

The instrument was pretested using cognitive interviews [34] that applied the think-aloud technique, paraphrasing, and verbal probing. These interviews were conducted with experts from various disciplines, including Social Sciences, Sociology, Health Sciences, Medical Sciences, and Ethics, as well as with a small patient sample in Germany, in collaboration with the German Heart Foundation. The purpose was to evaluate the clarity and understanding of key concepts, questions, and terminology, which resulted in several iterations of suggestions for improving the questionnaire as well as the video explanations—especially the understandability of the explanatory videos regarding PRP and AI has been a crucial goal.

Further refinements were made to the wording, and terminology was adjusted accordingly. The different versions of the questionnaire were translated back and forth by professional translators and native speakers for each of the main languages spoken in the selected countries (Dutch, English, German, and Spanish). After a description of the survey, data handling and protection, consent, instructions, and socioeconomic data, including the MacArthur Scale of Subjective Social Status [35,36], the questionnaire consists of 5 parts.

The first part is the adjusted and updated version (2.0) of the Technology Readiness Index (TRI) [37], which “assesses people’s propensity to accept as well as use an innovative technology for the accomplishment of his or her goals in life” [37]. TRI 2.0 consists of 4 dimensions: optimism, innovativeness, insecurity, and discomfort. Optimism and innovativeness are seen as motivators of technology readiness, whereas discomfort and insecurity act as inhibitors. Individuals may experience both motivating and inhibiting sentiments toward technology. The TRI is widely applied and provides impulses for methodological or conceptual discussion across various fields [38-42]. While other instruments were available [43-45], the TRI 2.0 was considered the best fit for this complex and long survey due to its practicality, conciseness, and shorter length.

After TRI 2.0 follows a section with questions regarding the application of PRP (in the decision for ICD implantation) and a section with questions regarding the application of AI (in the decision for ICD implantation). This is followed by questions about ethical considerations on noninferiority trials (NITs) with a focus on mortality as a primary end point—this section is not part of the results of this paper and is going to be published separately. Every section is introduced by a short descriptive video and accompanying infographics explaining the main concept (PRP, AI, and NIT). The questionnaire and the explanatory videos are provided in Multimedia Appendices 1 and 2.

Most of the questions used a 5-point Likert-type scale ranging from “strongly disagree” to “strongly agree,” except sociodemographic questions, 1 conditional question, and some open questions. The questionnaires were web-based and integrated into the platform LimeSurvey (LimeSurvey GmbH).

### Inclusion Criteria

The target population comprised adults aged 18 years or older living in Austria, Belgium, Germany, the Netherlands, Spain, or the United Kingdom who met at least one of the following (self-reported) clinical criteria: heart failure, previous myocardial infarction, survived cardiac arrest, or currently carrying an ICD. Participation was open to all individuals with access to the survey link. This self-selected recruitment approach corresponds to an unrestricted web survey and results in a partial, nonprobability sample. The survey also captured some patients’ perspectives from Austria as well as Belgium, which were—after careful consideration—included in the broader reporting and analysis in this study.

Although the survey is part of the PROFID project, the patient sample differed from the main trial study population (PROFID EHRA [28]). It included individuals at an increased risk of SCD (ie, potentially eligible for an ICD implantation for primary or secondary prevention) and patients who are already carrying an ICD. This provides a holistic perspective, allowing patients who have been directly affected by the decision-making process—whether or not to receive an ICD—to gain a better understanding of the potential implications of involvement of advanced new technologies in this decision.

### Recruitment and Sampling Procedures

Given the differences in samples and timelines, direct recruitment through the clinical trials was neither planned nor feasible. Therefore, a tailored recruitment strategy was developed for the survey. Recruitment began in April 2025 (United Kingdom), June 2025 (Germany), July 2025 (Netherlands), and August 2025 (Spain), and ended in December 2025.

In line with recommendations for nonprobability sampling, recruitment was conducted through a broad multichannel strategy. The recruitment process was coordinated internally by a 6-member project team and continuously monitored throughout the data collection period.

Members of the target population were generally not approached directly; instead, contact was established through intermediaries and network partners. Patient associations, self-help groups, professional societies, and other organizations related to cardiovascular disease were contacted and asked to share the survey link within their networks and encourage participation. Organizations, initiatives, and Facebook (Meta Platforms, Inc) groups contacted are listed in the Multimedia Appendix 3.

### Data Analysis

All statistical analyses were conducted using IBM SPSS Statistics version 31.0.0.0 with the R plugin. Prior to analysis, the raw survey data were cleaned and screened for data quality. This process included checking implausible values, identifying potential outliers, and excluding cases showing indications of speeding or patterned response behavior. The total analytic sample consists of participants who completed at least the TRI. The inhibitor dimensions discomfort and insecurity were inversely coded before calculating the overall TRI score [37]. Missing data in the TRI section were not imputed. We considered the possibility that missingness in the TRI items was not random, particularly because the inhibitor dimensions, discomfort, and insecurity may be more sensitive or unpleasant for some respondents to answer. Therefore, the missingness mechanism may plausibly have been missing not at random. This also applies to the PRP and AI sections; missingness may plausibly be associated with unobserved attitudes, comprehension, or engagement regarding these topics. In addition, most items provided a “prefer not to say” response option. Therefore, among respondents who completed the survey, missing substantive responses do not simply reflect accidental item nonresponse, but may represent a decision not to provide an answer.

The exploratory factor analysis (EFA) and the regression analyses were conducted on a subset of the total analytic sample. This subset consists of participants who completed the survey. To identify the underlying structure of the survey instrument, EFA with oblique rotation was conducted. The resulting factors were evaluated regarding their reliability and construct validity to establish internally consistent and theoretically meaningful factors. To evaluate the internal consistency of the identified factors, reliability analyses were conducted separately for each factor. Cronbach α was calculated, and corrected item-total correlations as well as changes in Cronbach α following the deletion of individual items were examined. The selected factors were retained because they met both statistical and conceptual criteria: they showed acceptable internal consistency, were supported by the loading pattern as well as the communality, and represented theoretically meaningful dimensions of patient attitudes toward PRP and AI in medical decision-making. For the subsequent calculation of mean scores for the factors identified in the EFA, one missing item was tolerated for 4- and 5-item factors; for the 3-item factor, complete item responses were required.

The means of these factors and the mean of the overall TRI were compared between the 3 main country samples. Exploratory country comparisons were conducted using Welch 1-way ANOVA because group sizes were unequal and Levene tests indicated that homogeneity of variance could not be assumed for all variables. Games-Howell tests were used for post hoc pairwise comparisons [46]. These analyses were restricted to Germany, the Netherlands, and the United Kingdom. The combined “Other” category was excluded because of its small and heterogeneous composition. Because the study was exploratory and hypothesis-generating, Benjamini-Hochberg false discovery rate (FDR) correction was applied as a sensitivity analysis to conceptually defined families of inferential tests like the results of the Games-Howell post hoc comparisons and the main regression analysis [47]. Additionally, 2 *t* tests (both 2-tailed) were conducted: a paired-samples *t* test comparing participants’ support for delegating ICD implantation decisions to PRP models versus AI, and an independent-samples *t* test comparing perceived AI benefits between men and women.

Regression analyses were performed with the subsample to examine the extent to which these factors, together with selected sociodemographic and socioeconomic characteristics, were associated with patients’ perspectives on PRP and the use of AI in ICD implantation. Regression assumptions were assessed through visual inspection of residuals and a modified Breusch-Pagan test. In the presence of heteroscedasticity, HC3 robust SEs were used [46]. As a sensitivity analysis, we applied FDR to the combined results of both main regression models. The primary regression models were repeated as an additional sensitivity analysis with the TRI subdimensions and with country dummy variables for the 3 main country samples. Germany was used as the reference category because it represented the largest country sample. The Netherlands and the United Kingdom were included as dummy variables. The combined “Other” category was also excluded from this sensitivity analysis.

### Ethical Considerations

The PROFID Patient Survey Study was reviewed by the Ethics Committee of the University of Bayreuth, Germany. The committee granted formal ethics approval (proposal number 24‐029). All participants were informed about the study objectives and procedures and provided informed consent at the beginning of the survey. Material for informed consent can be found in Multimedia Appendix 4. All participants have been informed about their right to withdraw at any time. Participation was anonymous, voluntary, and not compensated. Participants were asked not to deliver direct personal identifiable information. Upon collection, all data were anonymized and stored on secure, password-protected servers that could only be accessed by the responsible research team members.

## Results

### Overview

Across all countries, 764 respondents accessed the survey. Of these, 98 (12.8%) did not start the questionnaire or did not provide consent. In total, 666 (87.2%) participants began the survey. During data cleaning, respondents were excluded if they dropped out before completing the TRI section (n=79), failed to meet the inclusion criteria (n=43), provided incomplete personal information (n=15), or did not reside in the target countries (n=9).

Overall, 520 respondents completed the TRI section of the survey, corresponding to a completion rate of 78.1% (520/666) among those who started the survey. During quality assessment, we additionally excluded participants with missing TRI values (n=29) and cases identified as pattern or speeding responses (n=21). The final analytic sample consisted of 470 participants across all countries. Therefore, 5.8% (29/499) of the responses in the TRI section were excluded because of missing data. This corresponds to an effective completion rate of the TRI of 70.6% (470/666) among those who started the survey and were subsequently included in the analysis. The total completion rate—and therefore the subsample for the EFA as well as the regression analyses—of the survey was 53.2% (354/666), and the median duration was 21 (IQR 15-31) minutes.

The final sample consisted of 470 participants from Germany (n=210), the Netherlands (n=86), the United Kingdom (n=145), and 3 other European countries (n=29; Austria, Belgium, and Spain). Overall, 51.9% (244/470) of respondents were male and 48.1% (226/470) were female (Table 1). Participants were predominantly older adults: 31.3% (147/470) were aged 55‐64 years, 28.5% (134/470) were aged 65‐74 years, and 13.6% (64/470) were aged 75 years or older, whereas 9.8% (46/470) were aged 18‐44 years. The mean age of the sample was 61.12 (SD 12.62) years. Most respondents reported a history of cardiac conditions, including heart failure (69.1%, 325/470), myocardial infarction (37.9%, 178/470), and cardiac arrest (33%, 155/470). Regarding device therapy, 44.1% (207/470) had an ICD or S-ICD (subcutaneous implantable cardioverter defibrillator) implanted, 8.5% (40/470) had a CRT-D (cardiac resynchronization therapy defibrillator) device implanted, 3.2% (15/470) had an ICD or CRT-D device implanted, but were unsure about the device type, and 44.3% (208/470) had no device implanted. In terms of educational attainment, the largest groups reported vocational qualifications (27.4%, 129/470) or postgraduate degrees (24.5%, 115/470), followed by undergraduate degrees (20%, 94/470).

**Table 1.** Sample characteristics^a^.

Variable	Germany, n (%)	Netherlands, n (%)	United Kingdom, n (%)	Other^b^, n (%)	Total, n (%)
Population	210 (—^c^)	86 (—)	145 (—)	29 (—)	470 (—)
Gender					
Men	125 (59.5)	41 (47.7)	64 (44.1)	14 (48.3)	244 (51.9)
Women	85 (40.5)	45 (52.3)	81 (55.9)	15 (51.7)	226 (48.1)
Age (years)					
18‐44	15 (7.1)	9 (10.5)	15 (10.3)	7 (24.1)	46 (9.8)
45‐54	35 (16.7)	14 (16.3)	22 (15.2)	8 (27.6)	79 (16.8)
55‐64	57 (27.1)	33 (38.4)	53 (36.6)	4 (13.8)	147 (31.3)
65‐74	62 (29.5)	24 (27.9)	41 (28.3)	7 (24.1)	134 (28.5)
>75	41 (19.5)	6 (7)	14 (9.7)	3 (10.3)	64 (13.6)
Cardiac history^d^					
Myocardial infarction	99 (47.1)	29 (33.7)	38 (26.2)	12 (41.4)	178 (37.9)
Heart failure	136 (64.8)	66 (76.7)	99 (68.3)	24 (82.8)	325 (69.1)
Cardiac arrest	62 (29.5)	40 (46.5)	47 (32.4)	6 (20.7)	155 (33)
ICD^e^ implanted					
ICDor S-ICD^f^	65 (31)	58 (67.4)	70 (48.3)	14 (48.3)	207 (44)
CRT-D^g^	13 (6.2)	13 (15.1)	13 (9)	1 (3.4)	40 (8.5)
Unsure about device type	7 (3.3)	4 (4.7)	3 (2.1)	1 (3.4)	15 (3.2)
No	125 (59.5)	11 (12.8)	59 (40.7)	13 (44.8)	208 (44.3)
Educational attainment					
GCSEs^h^ (or equivalent)	33 (15.7)	20 (23.3)	25 (17.2)	8 (27.6)	86 (18.3)
A-Levels (or equivalent)	30 (14.3)	2 (2.3)	7 (4.8)	1 (3.4)	40 (8.5)
Vocational qualification	48 (22.9)	38 (44.2)	33 (22.8)	10 (34.5)	129 (27.4)
Undergraduate degree	24 (11.4)	16 (18.6)	48 (33.1)	6 (20.7)	94 (20)
Postgraduate degree	72 (34.3)	9 (10.5)	30 (20.7)	4 (13.8)	115 (24.5)
Employment status					
Employed or self-employed	79 (37.6)	39 (45.3)	42 (29)	12 (41.4)	172 (36.6)
Not employed	3 (1.4)	13 (15.1)	15 (10.3)	2 (6.9)	33 (7)
Student or in training	1 (0.5)	—	4 (2.8)	—	5 (1.1)
Retired	119 (56.7)	29 (33.7)	79 (54.5)	10 (34.5)	237 (50.4)
Unable to work	4 (1.9)	3 (3.5)	2 (1.4)	4 (13.8)	13 (2.8)
Prefer not to say	4 (1.9)	2 (2.3)	3 (2.1)	1 (3.4)	10 (2.1)
Subjective social status					
Low (1-4)	29 (13.8)	7 (8.1)	29 (20)	4 (13.8)	69 (14.7)
Medium (5-7)	149 (71)	48 (55.8)	86 (59.3)	24 (82.8)	307 (65.3)
High (8-10)	32 (15.2)	31 (36)	30 (20.7)	1 (3.4)	94 (20)

a The subset characteristics can be found in Multimedia Appendix 5.

b Other countries: Austria (n=8); Belgium (n=7); Spain (n=14).

c Not applicable.

d Multiple answers possible.

e ICD: implantable cardioverter-defibrillator.

f S-ICD: Subcutaneous Implantable Cardioverter Defibrillator.

g CRT-D: cardiac resynchronization therapy defibrillator.

h GCSE: General Certificate of Secondary Education.

### EFA

An EFA was conducted on 26 items of the thematic sections PRP and AI. One item showed a very low measure of sampling adequacy (MSA=0.35) and did not load substantially on any factor. The item was therefore removed prior to further factor analysis. Following inspection of the rotated solution, 2 further items were removed due to low communalities and weak factor loadings. The final EFA therefore included 23 items. Because correlations between factors were expected, an oblique rotation was applied [48]. The EFA excluded 4% (14/354) of responses in the subset due to missing data.

The Kaiser-Meyer-Olkin measure of sampling adequacy was 0.844, indicating meritorious sampling adequacy [49]. All individual MSAs exceeded 0.70, well above the recommended minimum of 0.50 [50]. Bartlett test of sphericity was significant (*χ*²_253_=4352.44, *P*<.001), indicating that the correlation matrix was suitable for factor analysis. An initial extraction was conducted to examine eigenvalues. Parallel analysis was used to determine the number of factors to retain (1,000 iterations), which is considered a more accurate method than relying solely on eigenvalues greater than 1 [51,52]. A 6-factor solution was indicated and therefore retained and interpreted (Table 2).

**Table 2.** Exploratory factor analysis results (n=340).

Factors^a^ and items	Communality (h^2^)	Factor loading
Perceived benefits and support for usage of PRP^b^ in MDM^c^		
I believe personalised risk prediction models improve the accuracy of medical diagnoses.	0.757	0.872
I believe personalised risk prediction models improve the accuracy of medical treatment decisions.	0.735	0.834
Health care providers should use personalised risk prediction models to support the decision whether to implant an ICD^d^ (S-ICD^e^/CRT-D^f^).	0.619	0.565
Personalised risk prediction models should support treatment decisions of health care providers.	0.468	0.542
Perceived benefits and support for usage of AI in MDM		
I believe AI can improve the accuracy of medical diagnoses.	0.757	0.834
I believe AI can improve the accuracy of medical treatment decisions.	0.723	0.828
AI should be used to support treatment decisions.	0.675	0.762
Health care providers should use AI to support the decision whether to implant an ICD (S-ICD/CRT-D).	0.494	0.687
Recommendations made by AI should complement the role of health care providers in treatment decisions.	0.491	0.659
Transparency expectations in algorithmic decision-making		
I want to be informed about how exactly AI is used in making my treatment decisions.	0.620	0.843
I want to know if AI is involved in my treatment decisions.	0.648	0.808
I want to be informed how exactly the personalised risk prediction model is used to influence my treatment decisions.	0.533	0.590
I want to know if a personalised risk prediction model is involved in my treatment decisions.	0.546	0.521
Support for delegation of decisions to algorithms		
Risk prediction models should make the decision whether to implant an ICD (S-ICD/CRT-D).	0.578	0.745
Health care providers should base their decision whether to implant an ICD (S-ICD/CRT-D) on risk prediction models.	0.683	0.733
AI should make the decision whether to implant an ICD (S-ICD/CRT-D).	0.414	0.601
Health care providers should base their decision whether to implant an ICD (S-ICD/CRT-D) on AI.	0.494	0.532
Self-reported AI literacy		
In my view, I have a good understanding of AI in general.	0.560	0.79
How often do you use applications based on AI technology in your daily life?	0.703	0.782
I am interested in AI.	0.654	0.629
Preference for shared decision-making		
I want to take part in the decision of whether to implant an ICD (S-ICD/CRT-D).	0.757	0.92
I want to take part in the decision of whether to implant an ICD (S-ICD/CRT-D), if a personalised risk prediction model is used.	0.696	0.812
I want to take part in the decision of whether to implant an ICD (S-ICD/CRT-D), if AI is used.	0.535	0.594

a Extraction method: principal axis factoring. Rotation method: direct oblimin with Kaiser normalization. Factor loadings are based on the pattern matrix. The rotation converged in 9 iterations. Listwise deletion.

b PRP: personalized risk prediction.

c MDM: medical decision-making.

d ICD: implantable cardioverter defibrillator.

e S-ICD: subcutaneous implantable cardioverter defibrillator.

f CRT-D: cardiac resynchronization therapy defibrillator.

The 6 factors together accounted for 61.9% of the total variance. The rotated solution revealed a clear and interpretable structure, with strong primary loadings and no substantial cross-loadings. Factor correlations were moderate, supporting the use of oblique rotation (Multimedia Appendix 5).

The first factor captured perceived benefits and support of PRP models in medical decision-making (MDM), indicated by items reflecting positive evaluations of such models in supporting clinical decisions. The second factor represented perceived benefits and support of AI in MDM, as indicated by strong loadings of items reflecting beliefs that AI improves diagnostic accuracy and should support treatment decisions. The third factor captured transparency expectations in algorithmic decision-making, comprising items expressing the desire to be informed about whether and how algorithmic systems are involved in treatment decisions. The fourth factor reflected support for delegating decisions to algorithms, including items endorsing the delegation of clinical decision-making authority to AI or risk prediction models. The fifth factor represented self-reported AI literacy, defined by items assessing familiarity with AI technologies due to a self-evaluation reported by items reflecting understanding, usage, and interest. This perceived literacy does not represent actual comprehension and reflected behavior. Finally, the sixth factor reflected the preference for SDM in clinical decisions, characterized by items expressing the desire to actively participate in treatment decisions.

All 6 factors demonstrated acceptable to excellent internal consistency, with Cronbach α coefficients ranging from 0.76 to 0.90. Corrected item-total correlations were satisfactory across all factors, indicating adequate homogeneity of the items within each construct. In addition, the removal of any individual item did not result in a meaningful improvement in reliability, supporting the retention of all items on their respective factors (Multimedia Appendix 5). Correlations among factors provided additional evidence of convergent and discriminant validity. Intercorrelations among the 6 factors ranged from *r*=−0.06 to *r*=0.55. Moderate correlations were observed between conceptually related constructs (eg, perceived benefits of AI and perceived benefits of PRP, *r*=0.55, *P*<.001), whereas other associations were weak, supporting the discriminant validity of the identified dimensions (Multimedia Appendix 5).

Table 3 presents descriptive statistics for all study factors across the main 3 country samples. The means of all singular items used in the EFA can be found in the Multimedia Appendix 5. Overall, perceived benefits of PRP, perceived benefits of AI, transparency expectations, and preferences for SDM were relatively high in all countries, whereas support for delegation of decisions to algorithms was lower.

**Table 3.** Reliability, variance explained, and descriptive statistics of the study factors by country.

Scale	Cronbach α^a^	Variance explained^a^ (%)	Germany, mean^b^ (SD)	Netherlands, mean^b^ (SD)	United Kingdom, mean^b^ (SD)
EFA^c^ factors					
Perceived benefits and support for usage of PRP^d^ in MDM^e^	0.85	4.41	4.33 (0.57)	4.11 (0.58)	4.10 (0.61)
Perceived benefits and support for usage of AI in MDM	0.90	27.69	4.08 (0.68)	3.79 (0.66)	3.76 (0.8)
Transparency expectations in algorithmic decision-making	0.84	12.41	4.50 (0.5)	4.21 (0.65)	4.50 (0.47)
Support for delegation of decisions to algorithms	0.76	8.84	2.63 (0.8)	2.84 (0.75)	2.56 (0.78)
Self-reported AI literacy	0.80	4.77	3.15 (0.84)	3.27 (0.88)	3.20 (0.88)
Preference for shared decision-making	0.84	3.78	4.58 (0.56)	4.32 (0.68)	4.50 (0.61)
TRI^f^	—^g^	—	3.19 (0.56)	3.38 (0.52)	3.06 (0.6)

a Alpha and explained variance were obtained from the entire population.

b Sample sizes for means (SDs) differ across factors due to missing responses. Germany (n=175‐210); Netherlands (n=58‐86); United Kingdom (n=105‐145). Full descriptive statistics can be found in Multimedia Appendix 5.

c EFA: exploratory factor analysis.

d PRP: personalized risk prediction.

e MDM: medical decision-making.

f TRI: Technology Readiness Index.

g Not applicable.

Games-Howell post hoc comparisons indicated that participants from Germany reported higher perceived benefits and support for PRP than participants from the Netherlands (*P*=.024) and the United Kingdom (*P*=.003), and higher perceived benefits and support for AI than participants from the Netherlands (*P*=.014) and the United Kingdom (*P*=.002). Transparency expectations were lower in the Netherlands than in Germany (*P*=.007) and the United Kingdom (*P*=.010), whereas preference for SDM was higher in Germany than in the Netherlands (*P*=.021). Technology readiness was highest in the Netherlands and significantly higher than in Germany (*P*=.013) and the United Kingdom (*P*<.001). No significant country differences were observed for self-reported AI literacy or support for delegation of decisions to algorithms. Effect sizes for the statistically significant country differences were small, with η² ranging from 0.026 to 0.045 for the significant country differences, and these country-level differences should therefore be interpreted cautiously. After FDR, country differences remained significant for perceived benefits and support of PRP, perceived benefits and support of AI, transparency expectations, preference for SDM, and the TRI score. The Welch ANOVA results and Games-Howell post hoc comparisons are reported in Multimedia Appendix 5.

### Additional Comparative Tests

A paired-samples *t* test was conducted to compare participants’ support for the delegation of ICD implantation decisions to PRP models versus AI (n=355). Support was significantly higher for delegation to PRP models (mean 3.13, SD 1.05) than for delegation to AI (mean 2.18, SD 0.77; *t*_354_=19.08; *P*<.001). The mean difference was 0.95 (95% CI 0.85‐1.04), indicating a large effect (Cohen *d*=1.01) according to Cohen conventions [53].

An independent-samples *t* test was conducted to compare perceived AI benefits between men (n=190) and women (n=165). Men reported significantly higher AI benefit scores (mean 4.07, SD 0.66) than women (mean 3.77, SD 0.76; *t*_353_=4.03; *P*<.001). The mean difference was −0.30 (95% CI 0.16‐0.45), corresponding to a small-to-moderate effect size (Cohen *d*=0.43). In an additional adjusted regression model (Multimedia Appendix 5), the previously observed gender difference in perceived AI benefits was attenuated and no longer statistically significant (β=−0.125, *P*=.102) after controlling for age, educational attainment, and self-reported AI literacy. Self-reported AI literacy remained the strongest factor (β=0.370, *P*<.001), whereas age showed a statistically significant but small positive association (β=0.009, *P*=.006).

### Regression Analysis

Multiple linear regression analysis was conducted to examine whether or how technological readiness, self-reported AI literacy, support for delegation of decisions to algorithms, transparency expectations in algorithmic decision-making, preferences for SDM, and educational attainment are associated with perceived benefits and support of PRP or AI in MDM. The regressions excluded 4% (14/354) and 3.7% (13/354) of responses in the subset due to missing data. The results are shown in Tables 4 and 5, respectively.

**Table 4.** Multiple linear regression model for perceived support and benefits of PRP^a^ in MDM^b^ (n=340).

Coefficient	β	rSE^c^^,^^d^	*t* statistic	95% CI	*P* value
(constant)	0.782	0.353	2.215	0.088 to 1.476	.027
TRI^e^ score	0.123	0.065	1.895	−0.005 to 0.251	.059
Self-reported AI literacy	0.094	0.050	1.896	−0.004 to 0.192	.059
Delegation of decisions to algorithms	0.265	0.047	5.676	0.173 to 0.357	<.001
Transparency in algorithmic decision-making	0.273	0.064	4.267	0.147 to 0.399	<.001
Shared decision-making	0.119	0.065	1.832	−0.009 to 0.246	.068
Educational attainment	0.044	0.025	1.741	−0.006 to 0.093	.083
Gender^f^	0.015	0.061	0.247	−0.105 to 0.135	.805
Age	0.003	0.003	1.015	−0.002 to 0.008	.311

a PRP: personalized risk prediction.

b MDM: medical decision-making.

c HC3-Method.

d rSE: robust SE.

e TRI: Technology Readiness Index.

f Gender coded as men=0 and women=1.

**Table 5.** Multiple linear regression model for perceived support and benefits of AI in MDM^a^ (n=341).

Coefficient	β	rSE^b^^,^^c^	t statistic	95% CI	*P* value
(constant)	0.217	0.451	0.481	−0.671 to 1.105	.631
TRI^d^ score	0.240	0.086	2.773	0.070 to 0.410	.006
Self-reported AI literacy	0.219	0.065	3.366	0.091 to 0.347	<.001
Delegation of decisions to algorithms	0.180	0.041	4.356	0.099 to 0.261	<.001
Transparency in algorithmic decision-making	0.180	0.067	2.694	0.048 to 0.311	.007
Shared decision-making	0.107	0.067	1.581	−0.026 to 0.239	.115
Educational attainment	0.052	0.026	2.049	0.002 to 0.102	.041
Gender^e^	−0.150	0.075	−1.998	−0.298 to −0.002	.047
Age	0.006	0.003	2.176	0.001 to 0.012	.030

a MDM: medical decision-making.

b HC3-Method.

c rSE: robust SE.

d TRI: Technology Readiness Index.

e Gender coded as men=0 and women=1.

The overall model was significant (*F*_8,331_=18.93, *P*<.001), explaining 31.4% of the variance in perceived PRP benefits (*R*²=0.314; adjusted *R*²=0.297). The residuals showed some deviation from normality. The modified Breusch-Pagan test indicated significant heteroscedasticity (*χ*²_1_=8.14, *P*<.004). In the robust model, support for delegation of decisions to algorithms (β=0.265, *P*<.001) and transparency expectations (β=0.273, *P*<.001) remained significant factors of perceived benefits of PRP in MDM. The results stayed robust after FDR.

The regression model associated with perceived benefits of AI in MDM was statistically significant, *F*_8,332_=21.15, *P*<.001, explaining 33.8% of the variance (*R*²=0.338; adjusted *R*²=0.322). The residuals showed some deviation from normality. The modified Breusch-Pagan test indicated significant heteroscedasticity (*χ*²_1_=17.16, *P*<.001). Using HC3 robust SEs, higher technological readiness (β=0.240, *P*=.006), self-reported AI literacy (β=0.219, *P*<.001), stronger support for delegating decisions to algorithms (β=0.180, *P*<.001), and higher transparency expectations (β=0.180, *P*=.007) associated with greater perceived benefits of AI in MDM. Educational attainment (β=0.052, *P*=.041), gender (β=−0.150, *P*=.047), and age (β=0.006, *P*=.030) also showed statistically significant associations. After FDR, the associations with technological readiness, self-reported AI literacy, support for delegating decisions to algorithms, and transparency expectations remained robust, whereas educational attainment, gender, and age were no longer considered robust (Multimedia Appendix 5).

In country-adjusted sensitivity analyses, the main substantive associations remained largely stable (Multimedia Appendix 5). In both regression models, participants from the Netherlands and the United Kingdom reported lower perceived support and benefits of PRP or AI than participants from Germany after adjustment for country. The inclusion of country dummies did not change the main substantive interpretation of the models. Additionally, we could not derive conclusive k-means clusters based on the TRI dimensions, as reported by Parasuraman and Colby [37]. For this reason, the overall TRI score was used in the primary regression models and country-level mean comparisons as an established measure of general technology readiness. To provide additional context, exploratory regression analyses using the 4 TRI dimensions instead of the overall TRI score are reported in Multimedia Appendix 5.

## Discussion

### Principal Findings

This study systematically examined patients’ perspectives across several European countries and explored how technological views and general attitudes toward technology relate to the evaluation of PRP and AI-supported approaches in life-threatening cardiac conditions and in particular ICD implantation. The findings support several existing assumptions in this field while also highlighting additional aspects that should be considered when high-level technologies are used in decision-making processes related to ICD implantation.

### Changes in Perception of PRP and AI

Although AI is widely seen as having the potential to improve health care, ethical concerns and mistrust toward AI-enabled medicine remain present among both the public and health care professionals [9,13,54]. Recent research suggests that public perceptions of AI in health care have become more critical between 2022 and 2024, with perceived risks increasing during this period [55]. Women, older individuals, and people with lower educational attainment were found to have lower odds of shifting toward a more positive perception of AI [55].

One explanation for these changes may lie in the growing visibility and accessibility of AI in everyday life. Before the broader public availability of tools such as ChatGPT, AI often remained an abstract concept, making it difficult for many individuals to form concrete opinions. The release of generative AI tools in late 2022 made AI more tangible through direct interaction. As a result, perceptions may have become less diffuse and more experience-based. Research indicates that direct exposure to AI can reduce uncertainty and lead to more differentiated views, even if these are not necessarily more positive overall [55]. In this sample, self-reported AI literacy and perceived benefits and support for AI are positively related. The broader shift in public perception forms an important backdrop for understanding how patients evaluate the implementation of AI and AI-supported tools in health care—also in cardiology.

### PRP and AI in MDM for Life-Threatening Heart Disease

The indication for ICD implantation does not represent an abstract technological use case, but a concrete clinical decision associated with both potential survival benefits and possible burdens for the patient [56]. The attitudes reported in this study should therefore not be understood merely as general views on technology. Rather, they reflect perspectives formed in a specific medical decision-making context in which probabilistic risk estimates, personal values, and individual life circumstances intersect. At the same time, these findings should be understood as reflecting participants’ evaluations of imagined or remembered scenarios rather than direct accounts of real-time clinical encounters. In the context of ICD implantation, decision-making is often emotionally charged, relationally negotiated, and shaped by prior illness experiences, trust in clinicians, family involvement, and the timing of care. These aspects are particularly important because the decision to implant an ICD is highly preference-sensitive for patients due to the considerable trade-offs involved [57].

Previous research has shown that a nonnegligible minority of patients later express regret about the decision, which is both clinically and ethically relevant [57]. ICD implantation is often framed as a life-saving intervention, yet some patients retrospectively question whether it was the right decision. This suggests that ICD-related decision-making is not purely risk-based, but also fundamentally value-based [20,21]. Even highly accurate PRP and AI models may remain insufficient if they fail to account for patient values, such as concerns about shock anxiety [58,59], preferences regarding life extension versus quality of life [60,61], or fears related to device dependency [62]. Even if the reported sample is not meant to be statistically representative for all indications, the sociodemographic characteristics are closely aligned with the group of patients eligible for ICD implantation in age [63] and with the gender distribution typically observed in patients with heart failure [64].

**Figure 1.**
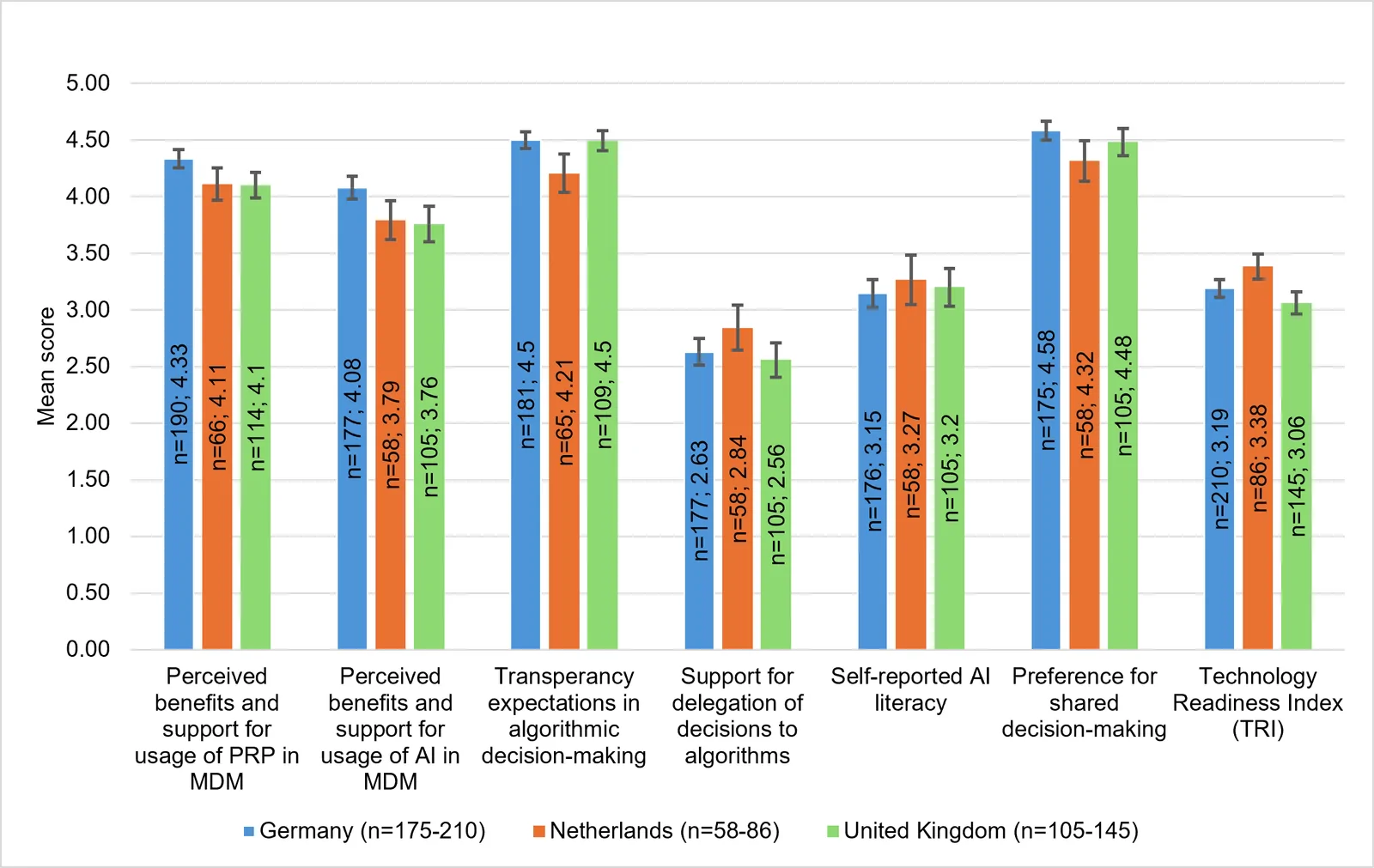
The means of exploratory factor analysis and means of TRI score for Germany, the Netherlands, and the United Kingdom (error bars indicate 95% CIs and full descriptive statistics can be found in Multimedia Appendix 5).

Against this background, PRP and AI were generally perceived by participants (refer to Tables 3–5; all individual items can be found in Multimedia Appendix 5) as useful tools to support decision-making regarding ICD indication (perceived benefits and support for usage of PRP in MDM and perceived benefits and support for usage of AI in MDM). As illustrated in Figure 1, the overall response patterns were broadly similar across Germany, the Netherlands, and the United Kingdom, although the magnitude of the factor means and TRI scores varied between countries. Based on comparative analyses (see “Additional Comparative Tests” section), participants reported significantly greater support for delegating ICD implantation decisions to models described as PRP than to models described as AI. This pattern is consistent with the broader finding that PRP was evaluated more favorably than AI across the sample. The distinction between the 2 concepts is a crucial point, especially given the fact that these 2 technologies are implemented in different combinations as well. One possible explanation is that PRP may have been perceived as more familiar and more directly linked to individualized care [65], whereas AI may have evoked broader associations with opacity, autonomy, or reduced human control [66,67]. Accordingly, the lower acceptance of AI should not be interpreted solely as a response to technical function, but also as reflecting the meanings and expectations attached to the term itself, even though both concepts were explained using extensively pretested visual and video-based materials (Multimedia Appendix 1). Most participants expressed support for their use, provided that transparency (see “Transparency Expectations in Algorithmic Decision-Making” section) is ensured. Patients also reported a very high preference for being actively involved in the decision-making process (preference for SDM) in ICD implantation, regardless of the use of PRP or AI. Therefore, this factor is not significantly associated with the perceived benefits and support of PRP and AI. Mandatory use and fully delegated decision-making to algorithmic systems were broadly rejected (see “Support for Delegation of Decisions to Algorithms” section). These patterns are largely consistent with findings from patient perspectives in other medical domains [68-71]. At the same time, PRP was viewed more favorably than AI across the sample.

### Transparency Expectations in Algorithmic Decision-Making

Transparency, defined as expressing the desire to be informed about whether and how algorithmic systems are involved in treatment decisions, emerged in this study as a central requirement for the patients’ acceptance of both PRP and AI-supported tools. Previous studies have shown that patients generally support the sharing and use of health data, provided that there is clarity about data control, access, and the purpose of use [14,72,73]. Conversely, lack of transparency substantially reduces support for the use of PRP and AI in health care [18]. Related to this, transparency appears to be shaped less by technical performance alone. It appears to be dependent on how systems are embedded in structures of accountability and communication. In this sense, transparency is important not only for building trust, but also for maintaining moral and legal (eg, AI Act) accountability [74-77].

The findings of these studies also point to a broader issue of responsibility. When algorithmic systems influence clinical recommendations, they may shift existing “role responsibilities” [78] and might create new responsibilities toward system designers, developers, and policymakers [6]. Yet it is patients who experience the downstream consequences of algorithmic error, despite having no control over the design of these systems. This tension becomes particularly relevant in medical contexts in which decisions involve high stakes and potential harm, for example, in ICD implantation.

Research further suggests that explanations to patients on the inclusion of (new) high technologies can have ambivalent effects. While they may increase confidence in a system, they can also make decision-making for caregivers feel more difficult by revealing tensions between algorithmic logic and clinical intuition [79]. Similarly, clinicians may become skeptical when AI recommendations deviate from guideline-based care or from individualized care practices. This indicates that successful implementation requires not only explainable systems, but also structures that support negotiation, interpretation, and context-sensitive use rather than rigid procedural compliance [79]—to ensure a transparent and even SDM process together with the patient.

### Support for Delegation of Decisions to Algorithms

The reported data showed that participants were significantly more supportive of delegating ICD implantation decisions to models perceived as PRP than to models perceived as based on AI (see “Additional Comparative Tests” section). Although PRP models were viewed more favorably in this context, this result should not be interpreted as an endorsement of independent algorithmic decision-making. Rather, the findings suggest higher general support for PRP models than AI, while decision delegation to algorithmic systems was still met with considerable skepticism overall (refer to Table 3 and Multimedia Appendix 5). Therefore, the results show very clearly that delegating decisions to algorithmic systems has been rejected and align with the findings of other studies [18,80].

In the context of ICD risk prediction, a nonexplainable high-risk (or low-risk) classification may be statistically valid, but if neither physician nor patient can adequately justify it, it remains ethically problematic [6]. Patients expect PRP and AI systems not only to perform well, but in many cases to exceed human accuracy, while simultaneously rejecting “invisible black box” systems [18,81]. Ultimately, there is a discrepancy between scientifically proven risks and their intuitive perception. This gap between statistical safety and patients’ perception can be attributed to a lack of trust—regarding clinicians using AI as well as merely “autonomous-like” AI systems [82].

### Sociodemographic Differences

The findings of the survey study should also be interpreted considering the specific sociodemographic variation of the target population (Table 1) in the acceptance of high technologies. Previous studies suggest that attitudes toward digital health tools in Germany differ according to age and gender [83]. More broadly, women, older individuals, and those with lower educational attainment appear less likely to develop more positive perceptions of AI over time [55]. These patterns regarding gender and education could also be observed in the reported study (see “Additional Comparative Tests” section). However, in an additional adjusted regression model controlling for age, educational attainment, and self-reported AI literacy, the gender coefficient was attenuated and no longer statistically significant. The regression model associated with perceived benefits and support of AI in MDM also showed significant influence of educational attainment, gender, and age. However, these findings could no longer be considered robust after FDR correction. This suggests that the unadjusted gender, age, and educational differences should be interpreted cautiously and may partly reflect differences in perceived AI familiarity or other aspects rather than sociodemographic influences alone. In contrast, the perceived benefits and support of PRP in MDM showed no significant differences (Table 4).

No substantial age-related differences were observed across the study scales. Although age was statistically associated with perceived benefits and support of AI in an extended regression model (Table 5), the estimated effect was very small: a 10-year increase in age corresponded to an estimated difference of approximately 0.06 points on the 5-point scale. Age was therefore not interpreted. This may be related to the special age characteristics of potential ICD patients (mean age 61.25, SD 12.62).

### Comparative International Perspectives

Although some cross-national differences were observed, the overall pattern of responses was broadly similar across Germany, the Netherlands, and the United Kingdom. Across all 3 countries, participants reported comparatively high support for personalized risk prediction models, high transparency expectations, and a strong preference for SDM, whereas support for delegating decisions to algorithms remained low. This suggests that attitudes toward AI-supported medical decision-making may be shaped less by national context alone than by more general expectations regarding patient involvement, transparency, and the appropriate role of technology in clinical care. At the same time, these broad similarities should be interpreted cautiously, as cultural expectations, health care system characteristics, public debates on digitalization, and differences in digital literacy were not modeled explicitly and therefore not interpreted directly.

Even though some differences may still be noteworthy. Participants from Germany, for example, tended to report somewhat more favorable attitudes toward both PRP models and AI in MDM, whereas respondents from the Netherlands showed slightly lower transparency expectations and higher technology readiness in general (Table 3). One possible interpretation is that cross-national differences in attitudes may reflect differences in the expected transparency and acceptance of algorithmic involvement in decision-making rather than differences in general technology readiness alone. This is consistent with the regression findings, which showed that the expected transparency and support for delegation of decisions to algorithms were among the strongest explanatory variables of perceived benefits and support for both PRP and AI.

Overall, the similarities across countries may be more important than the differences—even if it is possible that similar response patterns across countries conceal different contextual meanings or motivations. Comparable levels of support may therefore not necessarily indicate identical underlying understandings of PRP, AI, or (physician) responsibility in general. The findings indicate that, even in different European health care contexts, participants consistently favored a supportive rather than decisive role of algorithmic systems (Table 3). This leads to a potentially robust expectation that such systems should assist clinicians and patients but not replace human judgment or SDM. The successful implementation of AI in health care depends on its acceptance by key stakeholders, particularly patients, who are the primary beneficiaries of AI-driven outcomes [84].

### Patients’ Perspectives and the Human Dimension of Care

Placing a technology in a concrete clinical situation avoids overgeneralization and allows attitudes to be interpreted in relation to the actual decision at stake [82]. This is especially relevant in the case of patients’ perspectives on PRP models, AI-based systems, and combinations of PRP models and AI as decision support in ICD implantation, where different types may also evoke different expectations and concerns [9]. Predictive AI, generative AI, and increasingly agentic AI differ substantially in their functions, ranging from forecasting outcomes to producing content or acting on self-produced decisions toward defined objectives [85]. These distinctions are likely to become increasingly important for patients’ perceptions and should be communicated clearly. Previous research has identified several key attributes that shape patient views on AI-supported decision-making in health care: the type of AI decision, the level of explanation provided, performance or accuracy, responsibility for the final decision, the possibility of discrimination, and the severity of the disease context [86].

These findings are broadly in line with the presented framework and reinforce the need for differentiated, application-specific communication about PRP and AI systems in ICD implantation. The responses observed in this study should not be interpreted solely as expressions of acceptance or rejection of PRP and AI. They also point to broader expectations regarding how such technologies ought to be embedded in care. In particular, participants’ views appear to reflect concerns about transparency, responsibility, physician oversight, and the preservation of meaningful patient involvement. Lower acceptance of AI may therefore also indicate broader associations with opacity or loss of control, rather than a judgment of technical performance alone.

From a practical perspective, these findings suggest that transparency in clinical workflows should not be limited to the technical availability of information, but should include proactive communication with patients about whether PRP or AI-based tools as well as their combinations are being used, what their role in the decision process is, which kinds of data inform them, and where their limitations and uncertainties lie [87]. AI-supported recommendations should therefore be communicated as part of a physician-led conversation, embedded in SDM and open to questions, contextualization, and disagreement [16]. Algorithmic outputs should be communicated as one component of the decision-making process, not as a replacement for clinical judgment or patient preferences [88]. Clinical workflows should therefore include time and materials for explaining the system’s role, discussing uncertainty, and eliciting patient values [88]. For system designers, this implies that PRP and AI tools should generate outputs that are interpretable for clinicians and communicable to patients, including uncertainty estimates, plain-language summaries, and prompts for SDM rather than only risk scores or binary recommendations [89]. In this sense, implementation should support not only explainability at the system level, but also communicative practices that preserve patient involvement and clearly locate every step of responsibility of medical staff and institutions using technological systems [89,90].

In this regard, we highlight several findings that are very important in the context of a strong severity of consequences—such as empathetic flow of communications with the patient, SDM, and transparency toward the different use of high technology, especially on PRP models, AI-based systems, and combinations of PRP models and AI. The observed preference against full delegation of decision-making toward algorithmic systems also lends support to the right to a human physician proposed by Maris et al [21].

### Limitations

Several limitations should be considered when interpreting the findings. First, the self-selected online format likely favored participation by individuals with higher digital affinity, which may have increased reported technological openness and introduced self-selection and noncoverage bias [91]. In addition, recruitment through patient organizations, self-help groups, and disease-specific networks may have favored patients who were more engaged, informed, and proactive in managing their care, and who may therefore also have had higher health literacy, technological readiness, or a stronger preexisting interest in PRP and AI. The educational profile of the sample was relatively high, with undergraduate and postgraduate qualifications being overrepresented compared with the broader European population. As the study was based on a nonprobability sample and representativeness was not systematically assessed, the generalizability of the results is limited by these biases [92]. Second, there are some further limitations to report regarding the survey instrument and sample: (1) respondents with missing TRI values were excluded and missingness may have been related to unobserved technology-related discomfort or insecurity. Similarly, missingness in the PRP and AI sections may have affected the EFA-derived factor structure, factor means, and regression analyses if respondents with incomplete data differed systematically in their attitudes, comprehension, or engagement. However, the number of excluded cases was limited for both TRI values (29/499) and PRP and AI section items among EFA-eligible respondents (14/354). Potential bias due to missing data cannot be ruled out. (2) No technical measures such as cookies or IP checks were used to prevent duplicate entries, so repeated participation cannot be fully excluded. Moreover, (3) the completion rate suggests possible nonresponse bias, as participants who completed the survey may have differed systematically from those who dropped out. (4) The sample was dominated by participants from Germany, the Netherlands, and the United Kingdom, while numbers from the other countries were too small for meaningful country-specific comparison. Therefore, their answers have not been included in cross-country comparison. Third, the findings are closely tied to the specific context of ICD decision-making, a setting characterized by prognostic uncertainty, existential relevance, and value-based trade-offs. Attitudes toward transparency, delegation, and responsibility may therefore differ in other medical or technological contexts. In addition, actual ICD-related decision-making is often emotionally charged, interactive, and time-sensitive. The reported views should be understood as reflective attitudes rather than direct indicators of behavior in routine care. Even though PRP and AI were explained using extensively pretested multimedia materials and wording of items referring to those concepts, differences in how participants interpreted these concepts cannot be fully excluded. In this design, there was no focus on individuals who had decided against ICD implantation. As a result, their perspectives were not captured as a separate subgroup, although they may have provided important additional insights and a useful contrast to the present findings. Previous research has indicated that respondents who reported not wanting their ICD also recalled lower levels of participation in the decision-making process [57].

### Conclusion

In this multinational survey, patients generally supported the use of PRP models and AI-based systems as supportive tools in decision-making on ICD implantation, but not as substitutes for human judgment. Acceptance was closely tied to transparency, communication, technological readiness, and the preservation of patient involvement in decision-making. Across countries, participants consistently preferred a supportive rather than a decisive role for algorithmic systems, and PRP was viewed more favorably than AI in general.

Although the identified preferences cannot be directly transferred into ethical discourse, they offer important insights that, in combination with normative analysis, can help advance the debate on the ethically acceptable use of AI-based decision support systems. This study was situated in the context of SCD prevention and ICD implantation; its implications may be expected to extend beyond this specific clinical setting due to its design related to technological perceptions. The findings contribute to broader medical, ethical, and social debates on the use of PRP and AI in medicine by showing that the acceptability of such technologies is closely linked to how they are embedded in real-world care relationships, how clearly their purpose and limits are communicated, and whether patient values remain central to clinical decision-making.

## Supplementary material

10.2196/96968 Multimedia Appendix 1 Explanatory videos.

10.2196/96968 Multimedia Appendix 2 Promotional material.

10.2196/96968 Multimedia Appendix 3 Overview of all organizations contacted.

10.2196/96968 Multimedia Appendix 4 Informed consent and questionnaire including infographics.

10.2196/96968 Multimedia Appendix 5 Statistical calculations and quality assurance measures.

## Abbreviations

CRT-D: cardiac resynchronization therapy defibrillator
EFA: exploratory factor analysis
FDR: false discovery rate
ICD: implantable cardioverter defibrillator
MDM: medical decision-making
MI: myocardial infarction
MSA: measure of sampling adequacy
NIT: noninferiority trial
PROFID: Prevention of Sudden Cardiac Death After Myocardial Infarction by Defibrillator Implantation
PRP: personalized risk prediction
S-ICD: subcutaneous implantable cardioverter defibrillator
SCD: sudden cardiac death
SDM: shared decision-making
TRI: Technology Readiness Index
