# Supplementary material for: Patients’ Perspectives on Applications of AI and Personalized Medicine in Life-Threatening Heart Disease: European Cross-Sectional Patient Questionnaire Study
Source: J Med Internet Res. 2026 Aug 25;28:e96968. doi: 10.2196/96968 (PMC13505601; doi:10.2196/96968)
Supplement: Supplementary file 1 [file jmir-v28-e96968-s002.docx]

## Promotional Material

(1) **Flyer**

(2) **Poster**

All promotional materials are available in English, German, Spanish, and Dutch.
