# Supplementary material for: Patients’ Perspectives on Applications of AI and Personalized Medicine in Life-Threatening Heart Disease: European Cross-Sectional Patient Questionnaire Study
Source: J Med Internet Res. 2026 Aug 25;28:e96968. doi: 10.2196/96968 (PMC13505601; doi:10.2196/96968)
Supplement: Supplementary file 2 [file jmir-v28-e96968-s003.docx]

## Overview of all Organizations contacted

**Prioritization levels**

| **Code** | **Description** |
| --- | --- |
| **P1** | Top-level patient advocacy groups (national level; umbrella organization grouping national or regional org.; provide or partner with local support groups) **OR** local support groups with high representation of the target population (ICD, heart attack, heart insufficiency, sudden cardiac arrest) |
| **P2** | Top-level patient advocacy group or local support groups with focus on general and other heart diseases (e.g. congenital heart disease) |
| **P3** | Groups with limited thematic relevance to the target population, primarily included as potential indirect multipliers |
| **P4** | Groups not directly related to our target population (e.g. children, heart transplant, artificial heart) |
| **PS** | Medical specialty societies, (patient) charities dedicated to research |
| **PX_acronym** | The organization is affiliated with a higher-level organization |

**German Organizations**

| **Priority** | **Patient groups/organizations** | **Country** | **Specifics** | **Level** |
| --- | --- | --- | --- | --- |
| **1_DHS** | **Deutsche Herzstiftung (DHS)** | **Germany** | **General Heart Disease** | **National** |
| P1_dhs | "Leben mit einem Defi oder Herzschrittmacher" (Kassel) | Germany | Patients with ICD | Regional |
| P1_dhs | Defibrillator (ICD) SHG Hilden | Germany | Patients with ICD | Regional |
| P1_dhs | Defi-Gruppe RLP und Umgebung (Mainz) | Germany | Patients with ICD | Regional |
| P1_dhs | Herz- und Hirninfarkt SHG Jever | Germany | Heart attack | Regional |
| P1_dhs | Selbst-Hilfe-Gruppe "Herz-Helfer!" | Germany | Patients with ICD | Regional |
| P1_dhs | SHG Darmstadt: Leben mit Defi, Schrittmacher, Bypass, künstl. Herzklappe | Germany | Patients with ICD | Regional |
| P1_dhs | SHG Defi- und Telemedizin Eisenach | Germany | Patients with ICD | Regional |
| P1_dhs | SHG Herzinfarkt/Bypassoperierte/Herzklappenoperierte | Germany | Patients with ICD | Regional |
| P2_dhs | Berliner Powerherzen | Germany | Congenital Heart Disease | Regional |
| P2_dhs | Gesprächs- und SHG "Rund ums Herz" Siegburg | Germany | General Heart Disease | Regional |
| P2_dhs | Gesprächskreis Herz Bad Salzungen | Germany | General Heart Disease | Regional |
| P2_dhs | Gesprächskreis Herzkrankheiten (Wetzlar) | Germany | General Heart Disease | Regional |
| P2_dhs | Helfer fürs Herz Bremerhavener SHG | Germany | General Heart Disease | Regional |
| P2_dhs | Herzerkrankungen (Bottrop) | Germany | General Heart Disease | Regional |
| P2_dhs | Herz-Gesprächsgruppe Augsburg und Umgebung | Germany | General Heart Disease | Regional |
| P2_dhs | Herzgesprächsgruppe Düsseldorf | Germany | General Heart Disease | Regional |
| P2_dhs | Herz-Gesprächsgruppe Lübeck | Germany | General Heart Disease | Regional |
| P2_dhs | Herzgesprächskreis SHG Haren | Germany | General Heart Disease | Regional |
| P2_dhs | Herzgesundheit - SHG für Betroffene und Angehörige | Germany | General Heart Disease | Regional |
| P2_dhs | Herzgruppe Cloppenburg und umzu | Germany | General Heart Disease | Regional |
| P2_dhs | Herzpatienten im Gespräch (Bielefeld) | Germany | General Heart Disease | Regional |
| P2_dhs | Herz-Selbsthilfegruppe Arnsberg | Germany | General Heart Disease | Regional |
| P2_dhs | HERZ-SELBST-HILFE-GRUPPE Oldenburg (Hatten) | Germany | General Heart Disease | Regional |
| P2_dhs | Herz-SHG Aachen | Germany | General Heart Disease | Regional |
| P2_dhs | Herz-SHG Erftkreis | Germany | General Heart Disease | Regional |
| P2_dhs | Herz-SHG Gifhorn | Germany | General Heart Disease | Regional |
| P2_dhs | Herz-SHG im Landkreis Ebersberg | Germany | General Heart Disease | Regional |
| P2_dhs | Herz-SHG Oberberg | Germany | General Heart Disease | Regional |
| P2_dhs | Kulmbacher Koronare Selbsthilfe | Germany | General Heart Disease | Regional |
| P2_dhs | Beat Skipper SHG (online) | Germany | Arrhythmia | National |
| P2_dhs | SCAD-Herzen | Germany | SCAD | National |
| P2_dhs | Online-SHG Vorhofflimmern - Rund ums Herz | Germany | Atrial fibrillation | National |
| P2_dhs | Selbsthilfegruppe Herzpatienten Neustadt/Weinstr.-Haßloch und Umgebung | Germany | General Heart Disease | Regional |
| P2_dhs | SHG "Das Herz" (Hagen) | Germany | General Heart Disease | Regional |
| P2_dhs | SHG "Herz im Gespräch" und "Herzlich Willkommen" | Germany | General Heart Disease | Regional |
| P2_dhs | SHG "Herz und Seele" (Kassel) | Germany | General Heart Disease | Regional |
| P2_dhs | SHG "Herzgedanken" Heide | Germany | General Heart Disease | Regional |
| P2_dhs | SHG für Gerinnungs- und Herzklappenpatienten (Augsburg) | Germany | Heart valve disease | Regional |
| P2_dhs | SHG Gesprächskreis Herz Friedrichroda | Germany | General Heart Disease | Regional |
| P2_dhs | SHG HERZ Weilburg | Germany | General Heart Disease | Regional |
| P2_dhs | SHG Herz-Freizeit-Heilbronn | Germany | General Heart Disease | Regional |
| P2_dhs | SHG Herzklappe Hannover und Umgebung | Germany | Heart valve disease | Regional |
| P2_dhs | SHG Herzklappenerkrankungen (Bad Sooden-Allendorf) | Germany | Heart valve disease | Regional |
| P2_dhs | SHG Herz-Kreislauf-Erkrankte und deren Angehörige (Lemgo) | Germany | General Heart Disease | Regional |
| P2_dhs | SHG Herz-Kreislauferkrankungen Wilhelmshaven und Umgebung | Germany | General Heart Disease | Regional |
| P2_dhs | SHG Herzpatienten für Betroffene und Angehörige (Pirmasens) | Germany | General Heart Disease | Regional |
| P2_dhs | SHG Herzpatienten und Angehörige (Schwedt) | Germany | General Heart Disease | Regional |
| P2_dhs | SHG Herzpatienten Wiesbaden | Germany | General Heart Disease | Regional |
| P2_dhs | SHG im Raum Limburg-Diez | Germany | General Heart Disease | Regional |
| P2_dhs | SHG Paderborn für Herzkranke und u. ihre Angehörige (Paderborn) | Germany | General Heart Disease | Regional |
| P2_dhs | SHG Vorhofflimmern (Dresden) | Germany | Atrial fibrillation | Regional |
| P2_dhs | SHG Vorhofflimmern / Herzrhythmusstörungen (Berlin) | Germany | Atrial fibrillation | Regional |
| P2_dhs | SHG Vorhofflimmern Region Ulm und Neu-Ulm | Germany | Atrial fibrillation | Regional |
| P2_dhs | SHG Vorhofflimmern Speyer | Germany | Atrial fibrillation | Regional |
| P2_dhs | SHG Gesprächskreis (Eisenach) | Germany | General Heart Disease | Regional |
| **P1_HD** | **Herzschwäche Deutschland e.V. (HD)** | **Germany** | **Heart Failure** | **National** |
| P1_hd | Herzschwäche Deutschland e.V. – Nürnberg | Germany | Heart failure | Regional |
| P1_hd | Herzschwäche Deutschland e.V. – Rotenburg | Germany | Heart failure | Regional |
| **P2_HoS** | **Psychisch belastete Herzpatienten e. V. (Herz ohne Stress, HoS)** | **Germany** | **Mentally strained heart p.** | **National** |
| P2_hos | SHG Herzkrank ohne Ängste und Sorgen München | Germany | General Heart Disease | Regional |
| P1_hos | SHG Überlebter plötzlicher Herztod | Germany | Sudden cardiac arrest | Regional |
| P2_hos | Bonn SHG | Germany | General Heart Disease | Regional |
| P2_hos | EMAH-SHG Köln | Germany | Congenital Heart Disease | Regional |
| P2_hos | Herz-Gespräche am Montag Online | Germany | General Heart Disease | Regional |
| P2_hos | Ludwigshafen SHG | Germany | General Heart Disease | Regional |
| P2_hos | SHG FrauenHERZen in München | Germany | General Heart Disease | Regional |
| P2_hos | SHG für Angehörige in München | Germany | General Heart Disease | Regional |
| P2_hos | SHG Herz ohne Stress München | Germany | General Heart Disease | Regional |
| P2_hos | SHG in Berlin/Potsdam | Germany | General Heart Disease | Regional |
| P2_hos | SHG in der Klinik Barmelweid | Germany | General Heart Disease | Regional |
| P2_hos | SHG in Jena | Germany | General Heart Disease | Regional |
| P2_hos | SHG in Leipzig | Germany | General Heart Disease | Regional |
| P2_hos | SHG in Nürnberg | Germany | General Heart Disease | Regional |
| P2_hos | SHG in Regensburg | Germany | General Heart Disease | Regional |
| P2_hos | Völklingen SHG | Germany | General Heart Disease | Regional |
| **P1_ICD** | **Defibrillator (ICD) Deutschland e.V. (ICD)** | **Germany** | **Patients with ICD** | **National** |
| P1_icd | Defi-Selbsthilfegruppe Erlabrunn/Erzgebirge | Germany | Patients with ICD | Regional |
| P1_icd | Defi - Selbst – Hilfe – Gruppe – Freising | Germany | Patients with ICD | Regional |
| P1_icd | Defi - Selbsthilfegruppe Kempten | Germany | Patients with ICD | Regional |
| P1_icd | Defi - Selbsthilfegruppe München-Bogenhausen | Germany | Patients with ICD | Regional |
| P1_icd | Defi - SHG Bodensee- Oberschwaben (Ravensburg) | Germany | Patients with ICD | Regional |
| P1_icd | Defi - SHG Hochfranken/Hof | Germany | Patients with ICD | Regional |
| P1_icd | Defi (ICD) Selbsthilfegruppe Stuttgart-Ludwigsburg | Germany | Patients with ICD | Regional |
| P1_icd | Defi Gruppe Bremen e.V. | Germany | Patients with ICD | Regional |
| P1_icd | Defi Selbsthilfe Bad Rothenfelde | Germany | Patients with ICD | Regional |
| P1_icd | Defi Selbsthilfegruppe Harz | Germany | Patients with ICD | Regional |
| P1_icd | Defi Selbsthilfegruppe Heppenheim | Germany | Patients with ICD | Regional |
| P1_icd | Defi SHG Limburg Weilburg | Germany | Patients with ICD | Regional |
| P1_icd | Defi SHG´s Brandenburg | Germany | Patients with ICD | Regional |
| P1_icd | Defibrillanten Celle | Germany | Patients with ICD | Regional |
| P1_icd | Defibrillator Selbsthilfe Gruppe Oberfranken/Bayreuth | Germany | Patients with ICD | Regional |
| P1_icd | Defibrillator Selbsthilfe-Gruppe Pfaffenhofen a. d. Ilm | Germany | Patients with ICD | Regional |
| P1_icd | Defibrillator SHG Starnberg | Germany | Patients with ICD | Regional |
| P1_icd | Defi-Gruppe Euregio Klinik Nordhorn | Germany | Patients with ICD | Regional |
| P1_icd | Defi-Gruppe Westerwald-Siegerland, Siegen | Germany | Patients with ICD | Regional |
| P1_icd | Defihelden Wolfsburg | Germany | Patients with ICD | Regional |
| P1_icd | Defiherzen Erfurt | Germany | Patients with ICD | Regional |
| P1_icd | Defi-Selbsthilfe Rotenburg | Germany | Patients with ICD | Regional |
| P1_icd | Defi-Selbsthilfegruppe der Kerkhoff-Klinik in Bad Nauheim | Germany | Patients with ICD | Regional |
| P1_icd | Defi-Selbsthilfegruppe Eifel-Mosel | Germany | Patients with ICD | Regional |
| P1_icd | Defi-Selbsthilfegruppe in Heidelberg | Germany | Patients with ICD | Regional |
| P1_icd | Defi-Selbsthilfegruppe Leipzig und Umgebung | Germany | Patients with ICD | Regional |
| P1_icd | Defi-Selbsthilfegruppe München Deutsches Herzzentrum | Germany | Patients with ICD | Regional |
| P1_icd | Defi-Selbsthilfegruppe Reinbek | Germany | Patients with ICD | Regional |
| P1_icd | Defi-Selbsthilfegruppe Zwickau und Umgebung | Germany | Patients with ICD | Regional |
| P1_icd | Defi-Selbsthilfezirkel Kaiserslautern | Germany | Patients with ICD | Regional |
| P1_icd | Defi-SHG Kirchheim unter Teck | Germany | Patients with ICD | Regional |
| P1_icd | Defi SHG Bielefeld | Germany | Patients with ICD | Regional |
| P1_icd | Gruppe Kreis Höxter und Umland | Germany | Patients with ICD | Regional |
| P1_icd | HERZ IM TAKT SHG in Celle | Germany | Patients with ICD | Regional |
| P1_icd | ICD Kinder-/Jugend SHG Deutschland Süd | Germany | Patients with ICD | Regional |
| P1_icd | ICD- Selbsthilfegruppe Unterfranken | Germany | Patients with ICD | Regional |
| P1_icd | ICD-Selbsthilfegruppe Chemnitz u. Umgebung | Germany | Patients with ICD | Regional |
| P1_icd | ICD-Selbsthilfegruppe Marburg | Germany | Patients with ICD | Regional |
| P1_icd | Selbsthilfegruppe "Defibrillator" Lübben | Germany | Patients with ICD | Regional |
| P1_icd | Selbsthilfegruppe DEFI-Treff | Germany | Patients with ICD | Regional |
| P1_icd | Selbsthilfegruppe der Defi-Patienten Hamburg | Germany | Patients with ICD | Regional |
| P1_icd | Selbsthilfegruppe Wismar | Germany | Patients with ICD | Regional |
| P1_icd | SHG Halberstadt | Germany | Patients with ICD | Regional |
| P1_icd | SHG ICD Südbaden | Germany | Patients with ICD | Regional |
| P1_icd | SHG-Rostock im Cardio Consil | Germany | Patients with ICD | Regional |
| P1 | Deutsche Hochdruckliga e. V. | Germany | Hypertension | National |
| P2 | Herzgruppen Berlin (BGPR) | Germany | General heart disease | Regional |
| P1 | Herz in Takt Defi-Liga e.V. | Germany | Patients with ICD | National |
| P2 | ARVC-Selbsthilfe e. V. | Germany | Rare genetic heart disease | National |
| P2 | Bundesverein Jugendliche und Erwachsene mit angeborenem Herzfehler e.V. | Germany | Congenital Heart Disease | National |
| P2 | Förderkreis Herz- und Kreislaufhilfe e.V. | Germany | Charity for heart patients | Regional |
| P2 | Herzgruppen und Herzinsuffizienzgruppen im Saarland | Germany | General heart d. / exercise | Regional |
| P2 | Herzgruppen und Herzinsuffizienzgruppen in BaWü | Germany | General heart d. / exercise | Regional |
| P2 | Herz-LAG Bayern | Germany | General heart d. / exercise | Regional |
| P2 | Herzgruppen und Herzinsuffizienzgruppen in Bremen und Bremerhaven | Germany | General heart d. / exercise | Regional |
| P2 | Herzgruppen und Herzinsuffizienzgruppen in M-V | Germany | General heart d. / exercise | Regional |
| P2 | Herzgruppen und Herzinsuffizienzgruppen in Niedersachsen | Germany | General heart d. / exercise | Regional |
| P2 | Herzgruppen und Herzinsuffizienzgruppen in RLP | Germany | General heart d. / exercise | Regional |
| P2 | Herzgruppen und Herzinsuffizienzgruppen in Sachsen | Germany | General heart d. / exercise | Regional |
| P2 | Herzgruppen und Herzinsuffizienzgruppen in Sachsen-Anhalt und Thüringen | Germany | General heart d. / exercise | Regional |
| P2 | Herzgruppen und Herzinsuffizienzgruppen in Schleswig-Holstein | Germany | General heart d. / exercise | Regional |
| P2 | Herzwegweiser Berlin & Brandenburg e.V. | Germany | Online portal for support gr. | Regional |
| P2 | HOCM Deutschland e.V. | Germany | Hypertr. cardiomyopathy | National |
| P2 | SADS Deutschland e.V. | Germany | Sudden arrhythmic death sy. | National |
| P2 | Stiftung DHG (Diabetes Herz Gefäße) | Germany | P. with diabetes + heart d. | National |
| P4 | Bundesverband Herzkranke Kinder e.V. | Germany | Children/Congenital heart d. | National |
| P4 | HERZKIND e.V. | Germany | Children/Congenital heart d. | National |
| P4 | Kinderherzen heilen e.V. | Germany | Children/General heart d. | Regional |
| P4 | Kinderherzen Stiftung München | Germany | Children/Congenital heart d. | Regional |
| P4 | Pulslos Leben e.V. | Germany | Artificial Heart | National |

**Dutch Organizations**

| **Priority** | **Patient groups/organizations** | **Country** | **Specifics** | **Level** |
| --- | --- | --- | --- | --- |
| **P1_HS** | **Hartstichting (Dutch Heart Foundation)** | **Netherlands** | **Cardiovascular prevention & res.** | **National** |
| P1_hs | Harteraad (Dutch association for people with cardiovascular diseases) | Netherlands | Cardiovascular patient advocacy | National |
| P2_hs | Deltaplan Hartfalen | Netherlands | Heart failure care initiative | National |
| P2_hs | Hart4Onderzoek (Heart4Research, H4O) | Netherlands | Cardiovascular research funding | National |
| P3_hs | Hartveilig Drenthe | Netherlands | Cardiac safety and AED network | Regional |
| P3_hs | Reanimatie en AED NoordOost Overijssel | Netherlands | Resuscitation training and AEDs | Regional |
| P3_hs | HartsAlgNu | Netherlands | Acute cardiac symptom awareness | National |
| P4_hs | Stichting Hartedroom | Netherlands | Paediatric cardiomyopathy research | National |
| P1 | STIN (Foundation ICD Carriers Netherlands) | Netherlands | ICD patient support | National |
| P1 | Hartpatienten Nederland | Netherlands | Heart and vascular patients | National |
| P2 | Patientenvereniging Aangeboren Hartafwijkingen | Netherlands | Congenital heart disease | National |
| PS | Nederlandse Vereniging voor Cardiologie (NVVC) | Netherlands | Cardiology professional society | National |
| PS | Nederlandse Vereniging van Hart- en Vaatverpleegkundigen (NVHVV) | Netherlands | Cardiovascular nursing society | National |
| P2 | Hartgroup Nederland | Netherlands | Cardiac patient peer support | National |
| P2 | Belangenvereniging Hart- & Vaatpatiënten (BHV) | Netherlands | Patient advocacy network | National |
| P3 | Stichting PH Nederland | Netherlands | Pulmonary hypertension patients | National |
| P3 | Marfan Nederland | Netherlands | Marfan syndrome patients | National |
| P3 | Patientenfederatie | Netherlands | Patient organisation umbrella | National |
| P4 | Patiëntenvereniging Hart & Long Transplantatie | Netherlands | Heart and lung transplant patients | National |
| P3 | Hart in shape | Netherlands | Lifestyle support for heart patients | Regional |
| PS | Harten vaat onderzoekfonds Limburg | Netherlands | Cardiovascular research foundation | Regional |
| P3 | Noordwest ziekenhuisgroep | Netherlands | Hospital network for patient inform. | Regional |

**UK Organizations**

| **Priority** | **Patient Groups/organizations** | **Country** | **Specifics** | **Level** |
| --- | --- | --- | --- | --- |
| **P1_BHF** | **British Heart Foundation (BHF)** | **UK** | **General heart disease** | **National** |
| P1_bhf | Defibbers Cymru | UK | Patients with implanted ICD | Regional |
| P1_bhf | Essex Heartbeat - Support Group | UK | Patients with implanted ICD | Regional |
| P1_bhf | West Dorset ICD Support Group | UK | Patients with implanted ICD | Regional |
| P2_bhf | Active Hearts Wilmslow - Support Group | UK | General heart disease | Regional |
| P2_bhf | Ammanford Heart To Heart Support Group | UK | General heart disease | Regional |
| P2_bhf | Barnsley Coronary Club - Support Group | UK | General heart disease, exercise group | Regional |
| P2_bhf | Bedale Heart Support Group | UK | General heart disease, exercise group | Regional |
| P2_bhf | BHF Together Support Group Blackburn | UK | General heart disease | Regional |
| P2_bhf | BHF Together Support Group Carlisle | UK | General heart disease | Regional |
| P2_bhf | BHF Together Support Group Liverpool | UK | General heart disease | Regional |
| P2_bhf | BHF Together Support Group Selby | UK | General heart disease | Regional |
| P2_bhf | Bradford Heart Support Group | UK | General heart disease | Regional |
| P2_bhf | C.R.E.W. Heart Support Group | UK | General heart disease, exercise group | Regional |
| P2_bhf | Cardiac Friends Support Group | UK | General heart disease | Regional |
| P2_bhf | Charing Cross Cardiac Support Group | UK | General heart disease | Regional |
| P2_bhf | Crawley and Horsham Heart Support Group | UK | General heart disease | Regional |
| P2_bhf | Dicky Tickets Heart Support Group | UK | General heart disease | Regional |
| P2_bhf | Dronfield Friends Support Group | UK | General heart disease | Regional |
| P2_bhf | Durham Coronary Support Group | UK | General heart disease | Regional |
| P2_bhf | East Hants Heart Group | UK | General heart disease | Regional |
| P2_bhf | East Lindseay Heart Support Group | UK | General heart disease | Regional |
| P2_bhf | Exercise for the Heart | UK | General heart disease, exercise group | Regional |
| P2_bhf | Happy Hearts (Wales) Support Group | UK | General heart disease, exercise group | Regional |
| P2_bhf | Happy Hearts Exercise and Support Group | UK | General heart disease, exercise group | Regional |
| P2_bhf | Harrogate Heart Support Group | UK | General heart disease, exercise group | Regional |
| P2_bhf | Heart Link Support Group | UK | General heart disease, exercise group | Regional |
| P2_bhf | Heart to Heart Exercise and Support Group | UK | General heart disease, exercise group | Regional |
| P2_bhf | Heart2heart | UK | General heart disease, exercise group | Regional |
| P2_bhf | Heart2heart Support Group | UK | General heart disease, exercise group | Regional |
| P2_bhf | Heartbeat 95 | UK | General heart disease, exercise group | Regional |
| P2_bhf | Heartbeat Support Group Exeter | UK | General heart disease | Regional |
| P2_bhf | HeartBeat Support Woking | UK | General heart disease | Regional |
| P2_bhf | Heartbeat The Brighouse Heart Support Group | UK | General heart disease | Regional |
| P2_bhf | Heartbeats, The St. Albans and District Cardiac Support Group | UK | General heart disease | Regional |
| P2_bhf | HEARTLINK Ealing Hospital Heart Support Group | UK | General heart disease | Regional |
| P2_bhf | Lighthearted | UK | General heart disease, exercise group | Regional |
| P2_bhf | Midlleton Heartline Club | UK | General heart disease | Regional |
| P2_bhf | Milton Keynes Community Cardio-pulmonary Group | UK | General heart disease, exercise group | Regional |
| P2_bhf | Pontypool Pacers Heart Support Club | UK | Heart attack, heart surgery | Regional |
| P2_bhf | Poole Heart Support Group | UK | General heart disease | Regional |
| P2_bhf | Pulsations | UK | General heart disease, exercise group | Regional |
| P2_bhf | Restart Rotherham - Support Group | UK | General heart disease, exercise group | Regional |
| P2_bhf | Rochdale Heartbeat - Support Group | UK | General heart disease | Regional |
| P2_bhf | Salford Heart Care - Support Group | UK | General heart disease | Regional |
| P2_bhf | Sheffield Open Heart Club (SHOC) - Support Group | UK | Heart surgery | Regional |
| P2_bhf | St Austell Heart Support Group | UK | General heart disease, exercise group | Regional |
| P2_bhf | Stafford & Stoke-on-Trent Heart Support Group | UK | General heart disease | Regional |
| P2_bhf | Stockport Heart Support Group | UK | General heart disease | Regional |
| P2_bhf | Take Heart Leicester - Support Group | UK | General heart disease | Regional |
| P2_bhf | Take Heart Rugby - Support Group | UK | General heart disease, exercise group | Regional |
| P2_bhf | Take Heart Support Group | UK | General heart disease | Regional |
| P2_bhf | The Cardiac Exercise Club @SW19 | UK | General heart disease, exercise group | Regional |
| P2_bhf | The Cardiac Support Group Nottingham | UK | General heart disease | Regional |
| P2_bhf | The Sunderland Cardiac Support Group | UK | General heart disease | Regional |
| P2_bhf | Tickers Cardiac Support Group | UK | General heart disease, heart attack | Regional |
| P2_bhf | Trafford Strong Heart Support Group | UK | General heart disease, exercise group | Regional |
| P2_bhf | Washington Young at Heart - Support Group | UK | General heart disease, exercise group | Regional |
| P2_bhf | Wharfedale Cardiac Club - Support Group | UK | General heart disease, exercise group | Regional |
| P2_bhf | Wight Cardiac Rehab | UK | General heart disease, exercise group | Regional |
| P2_bhf | Wincanton Heart Support Group | UK | General heart disease, exercise group | Regional |
| P2_bhf | Winchester Heart Support Group | UK | General heart disease, exercise group | Regional |
| P2_bhf | Worcester Heart Support Group | UK | General heart disease | Regional |
| P2_bhf | Upbeat Heart Support | UK | General heart disease | Regional |
| **P1_CHSS** | **Chest Heart and Stroke Scotland (CHSS)** | **UK** | **General heart disease** | **National** |
| P2_chss | All Stroked Out | UK | General heart disease | Regional |
| P2_chss | Back into circulation | UK | General heart disease | Regional |
| P2_chss | Cairdeas, Lochalsh CHS | UK | General heart disease | Regional |
| P2_chss | Caithness Heart Support Group | UK | General heart disease | Regional |
| P2_chss | Carluke Cardiac Rehabilitation & Support Group | UK | General heart disease, exercise group | Regional |
| P2_chss | Clacks Council Active Communities | UK | General heart disease, exercise group | Regional |
| P2_chss | Crichton Hale & Hearty Club | UK | General heart disease, exercise group | Regional |
| P2_chss | D.D Hearties | UK | General heart disease, exercise group | Regional |
| P2_chss | Eyemouth & District Rehab Support Group | UK | General heart disease, exercise group | Regional |
| P2_chss | Fit over 50 | UK | General heart disease, exercise group | Regional |
| P2_chss | Foos Yer Doos Sing Song Club | UK | General heart disease | Regional |
| P2_chss | Gala Windbags | UK | General heart disease, exercise group | Regional |
| P2_chss | GCRA+ | UK | General heart disease, exercise group | Regional |
| P2_chss | GCRA+ (Banchory - Healthy Heart) | UK | General heart disease, exercise group | Regional |
| P2_chss | Glasgow West Heartbeats | UK | General heart disease, exercise group | Regional |
| P2_chss | Glenrothes Heart Support Group | UK | General heart disease, exercise group | Regional |
| P2_chss | GOGA Tayside Stretch & Relaxation | UK | General heart disease, exercise group | Regional |
| P2_chss | Grumpy Gaspers | UK | General heart disease | Regional |
| P2_chss | Health Hearts at Westwood | UK | General heart disease, exercise group | Regional |
| P2_chss | Heartbeat Cardiac Support Group | UK | General heart disease | Regional |
| P2_chss | Inverclyde Globetrotters | UK | General heart disease, exercise group | Regional |
| P2_chss | Inverness Bravehearts | UK | General heart disease, exercise group | Regional |
| P2_chss | Johnstonebridge Lunch Club | UK | General heart disease | Regional |
| P2_chss | Keep Ticking Falkirk | UK | General heart disease, exercise group | Regional |
| P2_chss | Killie Heartmates | UK | General heart disease, exercise group | Regional |
| P2_chss | Kirkcudbright Hale & Hearty Club | UK | General heart disease, exercise group | Regional |
| P2_chss | KPC Exercise Class | UK | General heart disease, exercise group | Regional |
| P2_chss | Live Well East Lothian | UK | General heart disease, exercise group | Regional |
| P2_chss | Lochwinhoch Hearties Keep Fit Group | UK | General heart disease, exercise group | Regional |
| P2_chss | Nairn Cardiactive Group | UK | General heart disease, exercise group | Regional |
| P2_chss | Orkney Geart Support Group | UK | General heart disease, exercise group | Regional |
| P2_chss | Pilton Equalities Project (PEP) | UK | General heart disease | Regional |
| P2_chss | Sit Fit @ The BLC and Community Hub | UK | General heart disease, exercise group | Regional |
| P2_chss | St Roch's Cardiac Support Group | UK | General heart disease, exercise group | Regional |
| P2_chss | Stirling Healthy Hearts Club | UK | General heart disease, exercise group | Regional |
| P2_chss | The Hoopers | UK | General heart disease, exercise group | Regional |
| P2_chss | The Hoopers - Pentland Community Centre | UK | General heart disease, exercise group | Regional |
| P2_chss | Walk Moray | UK | General heart disease, exercise group | Regional |
| P2_chss | Women's Health Support Group | UK | General heart disease | Regional |
| P2_chss | You're never too old | UK | General heart disease | Regional |
| P2_chss | Sallis Happy Hearts | UK | Cardiac Rehabilitation and Hearth Health | Regional |
| **P1_CHSS** | **British Society of Lifestyle Medicine** | **UK** | **General heart disease** | **National** |
| P1 | Arrythmia Alliance (AA) | UK | Arrythmias (coalition of charities, patients and patient groups, carers, medical groups and allied professionals) | National |
| P1 | Cardiomyopathy UK (CM) | UK | Cardiomyopathy, myocarditis | National |
| P1 | Heart Failure Warriors | UK | Heart Failure | Regional |
| P1 | Milton Keynes ICD support group | UK | Patients with implanted ICD | Regional |
| P1 | Northern Ireland Chest Heart and Stroke (NICHS) | UK | General heart disease | National |
| P1 | Pumping Marvellous Foundation | UK | Heart Failure | National |
| P1 | Somerville Heart Foundation | UK | Congenital heart disease | National |
| P1 | Sudden Cardiac Arrest UK | UK | Sudden Cardiac Arrest | National |
| P1 | The British Cardiac Patients Association | UK | General heart disease | National |
| P1 | ICD Online Support Group | UK | Patients with implanted ICD | National |
| P2 | Cardiac Rehab Support West Sussex | UK | General heart disease, exercise group | Regional |
| P2 | Heartbeat North West Cardiac Care | UK | General heart disease, exercise group | Regional |
| P2 | Norfolk Zipper Club | UK | Heart surgery | Regional |
| P4 | Children's Heart Foundation (CHF) | UK | Congenital heart disease, children | National |
| P4 | Children's Heartbeat Trust | UK | Congenital heart disease, children | Regional |
| PS | British Society for heart failure | UK | Heart failure, Charity of healthcare professionals | National |

**Spanish Organizations**

| **Priority** | **Patient Groups/organizations** | **Country** | **Specifics** | **Level** |
| --- | --- | --- | --- | --- |
| **P1_FEC** | **Fundacion Espanola del Corazon (Spanish Heart Foundation, FEC)** | **Spain** | **Spanish heart foundation, General Heart Disease** | **National** |
| P1_fec | Asociación de Trasplantados de Corazón e Insuficiencia Cardiaca de Andalucía (ATCICA) | Spain | Heart insufficiency, heart transplant | Regional |
| P2_fec | Adultos con Cardiopatías Congénitas de España Superando Obstáculos (ACCESO) | Spain | Congenital heart disease, adults | National |
| P2_fec | AEMCH (Asociacion espanola de miocardiopatie hipertrofica) | Spain | Hypertrophic Cardiomyopathy | National |
| P2_fec | AMICOR (Asociación Amigos del Corazón Área Sanitaria de Ferrol) | Spain | General heart disease | Regional |
| P2_fec | ASENCO (Asociación de Enfermos del Corazón Pulmón y otros Órganos Las Palmas) | Spain | General heart disease | Regional |
| P2_fec | Asociación Aragonesa Enfermos y Trasplantados de Corazón "Virgen del Pilar" (AATTCO) | Spain | General heart disease | Regional |
| P2_fec | Asociación Astorgana de Prevención Cardiaca (APRECA) | Spain | General heart disease, exercise group | Regional |
| P2_fec | Asociación Cardiaca "Trébol de Corazones" de Cádiz | Spain | General heart disease | Regional |
| P2_fec | Asociación Cardiópatas Valle-Kas (A.C.V.K.) | Spain | General heart disease | Regional |
| P2_fec | Asociación Cardiosaludable Madrid Sur (ACARMAS) | Spain | General heart disease | National |
| P2_fec | Asociación Corazón y Vida de Canarias | Spain | General heart disease | Regional |
| P2_fec | Asociación Corazones Sanos del Henares (CSdH) | Spain | General heart disease | National |
| P2_fec | Asociación de Anticoagulados de Aragón (ASANAR) | Spain | General heart disease | Regional |
| P2_fec | Asociación de Enfermos del Corazón de Almería y Provincia (AENCOAL) | Spain | General heart disease | Regional |
| P2_fec | Asociación de Pacientes Anticoagulados y Coronarios de Málaga (APAM) | Spain | General heart disease | Regional |
| P2_fec | Asociación de Pacientes Cardiacos de Córdoba y Provincia (ASPACACOR) | Spain | General heart disease | Regional |
| P2_fec | Asociación de Pacientes Cardiacos de Granada y su Provincia (ASPACGRAP) | Spain | General heart disease | Regional |
| P2_fec | Asociación de Pacientes Cardiacos de Jaén y Provincia (CARDIOJAÉN) | Spain | General heart disease | Regional |
| P2_fec | Asociación de Pacientes Cardiacos y Anticoagulados de Granada MUCHO CORAZÓN | Spain | General heart disease | Regional |
| P2_fec | Asociación de Pacientes Cardiacos y Anticoagulados Portuenses (ACAP) | Spain | General heart disease | Regional |
| P2_fec | Asociación de Pacientes Cardiópatas SUR SUM COR | Spain | General heart disease | Regional |
| P2_fec | Asociación de Pacientes de Rehabilitación Cardiaca de Aragón RE- LATE | Spain | General heart disease, exercise group | Regional |
| P2_fec | Asociación El PacienteXperto Salamanca | Spain | General heart disease | Regional |
| P2_fec | Asociación Española de Portadores de Válvulas Cardíacas, Anticoagulados y Adultos con Cardiopatías Congénitas (AEPOVAC) | Spain | General heart disease | National |
| P2_fec | Asociación Leonesa para la Prevención Cardiaca | Spain | General heart disease, exercise group | Regional |
| P2_fec | Asociación Nacional de Hipertensión Pulmonar (ANHP) | Spain | Pulmonary hypertension | National |
| P2_fec | Asociación Nuevo Camino para el Corazón Onubense (ANCCO) | Spain | General heart disease | Regional |
| P2_fec | Corazon sin fronteras | Spain | General heart disease | National |
| P2_fec | Federación Andaluza de Rehabilitados Cardiacos (FARCA) | Spain | General heart disease, exercise group | Regional |
| P2_fec | FEVASCAR (Federación Vasca de Pacientes Cardiovasculares ) | Spain | General heart disease | National |
| P2_fec | Fundación Contra la Hipertensión Pulmonar (FCHP) | Spain | Pulmonary hypertension | National |
| P2_fec | INSVACOR (Institut Valenciá del Cor) | Spain | General heart disease, rehab | Regional |
| P4_fec | ASEXTRAS (Asociación Extremeña de Trasplantados) | Spain | Heart transplant | Regional |
| P4_fec | Asociación Pretrasplante y Trasplantados de Castilla-La Mancha | Spain | Heart transplant | Regional |
| P4_fec | ATEHCNA (Asociación de Personas Enfermas y Trasplantadas de Hígado y Corazón de Navarra) | Spain | Heart transplant | Regional |
| P4_fec | AVATCOR (Asociación Valenciana de Trasplantados de Corazón) | Spain | Heart transplant | Regional |
| P4_fec | FETCO (Federación Española de Trasplantados de Corazón) | Spain | Heart transplant | National |
| **P1_CA** | **Cardioalianza (CA)** | **Spain** | **General Heart Disease, patient-led alliance representing cardiovascular patient organizations, focused on advocacy, empowerment, and self-management support** | **National** |
| P1_ca | SAMS (Síndromes Arrítmicos relacionados con la Muerte Súbita) | Spain | Arrythmias, sudden cardiac death | National |
| P2_ca | AACIC (Association of Congenital Heart Disease) | Spain | Congenital heart disease | National |
| P2_ca | ACPA (Asociación Cordobesa de Pacientes Anticoagulados) | Spain | Patients who take anticoagulants | Regional |
| P2_ca | amac (Asociación de Pacientes Anticoagulados & Cardiovasculares) | Spain | General Heart Disease | Regional |
| P2_ca | APACAM (Asociación ce cardiópatas y Anticoagulados de La Coruña) | Spain | Cardiomyopathies | Regional |
| P2_ca | APACOAL DEL ÁGUILA (Asociación de enefermos cardíacos del Águila de Alcalá ) | Spain | General heart disease | Regional |
| P2_ca | Asociación Orión, de Trasplantados, Donantes y Cardíacos de Algeciras | Spain | Transplant recipients, anticoagulated, cardiac, valve, baiypas, pacemakers | Regional |
| P2_ca | ASPACACA (Asociación de pacientes cardíacos de Camas y Aljarafe) | Spain | General heart disease | Regional |
| P2_ca | ASPACOR (Asociación de pacientes cardíacos de paradas) | Spain | General heart disease | Regional |
| P2_ca | Associació de Malalts Cardiacs i Anticoagulats | Spain | General heart disease | National |
| P2_ca | Canarios con corazón (Asociación de pacientes cardiacos coronarios anticoagulados y transplantados) | Spain | General heart disease | Regional |
| P2_ca | Catcecor (Associació de Pacients Cardiòpates de la Catalunya Central) | Spain | General heart disease | Regional |
| P2_ca | Fundacion Menudos Corazones - Menudos Corazones Foundation | Spain | Congenital heart disease | National |
| P2_ca | GICOR (Associació Gironina de Prevenció i Ajuda a les Malalties del Cor) | Spain | General heart disease | Regional |
| P2_ca | Todo corazon de murcia | Spain | Congenital heart disease | Regional |
| P4_ca | Asociación de Trasplantados de Corazón de Castilla y León | Spain | Heart transplant | Regional |
| P4_ca | Asociación Pequeños Corazones de Aragón | Spain | Congenital Heart Disease, children | Regional |
| P4_ca | Bihotzez (Asociación de ayuda a niños/as y jóvenes con cardiopatía congénita y sus familias) | Spain | Congenital Heart Disease, children | National |
| P4_ca | Corsnous (Associació Transplantates Cardíacs) | Spain | Heart transplant | National |
| **P2_CA_CS** | **Cardio Sevilla (Federacion de asociaciones del corazon de sevilla y provincia, CS)** | **Spain** | **General heart disease** | **Regional** |
| P2_ca_cs | ACARVISO (Asociacion cardiaca visuena) | Spain | General heart disease | Regional |
| P2_ca_cs | APACOR (Asociación Pacientes Coronarios) | Spain | General Heart Disease | National |
| P2_ca_cs | APEC (Asociación de pacientes cardíacos de Écija) | Spain | General heart disease | Regional |
| P2_ca_cs | ASANCOR (Asociación de pacientes coronarios nazarena) | Spain | General heart disease | Regional |
| P2_ca_cs | ASEVCOR (Asociación sevillana del corazón) | Spain | General heart disease | Regional |
| P2_ca_cs | Asociación de cardíacos de El Cuervo (El Cuervo Late) | Spain | General heart disease | Regional |
| P2_ca_cs | ASPACOM (Asociacion de pacientes coronarios de moron y su comarca) | Spain | General heart disease | Regional |
| P2_ca_cs | ASPACOMAR (Asociación de pacientes cardíacos de Marchena) | Spain | General heart disease | Regional |
| P2_ca_cs | ASUTRECOR (Asociacion utrerana del corazon) | Spain | General heart disease | Regional |
| P2_ca_cs | CARDIOALCOR (Asociación cardíaca de Mairena del Alcor) | Spain | General heart disease | Regional |
| P2_ca_cs | Corazones en camino | Spain | General heart disease | Regional |
| P2_ca_cs | MONTECOR (Asociación de pacientes cardíacos de Montellano y Coripe) | Spain | General heart disease | Regional |
| P2_ca_cs | PACOVAL (Asociación de pacientes coronarios de Los Palacios) | Spain | General heart disease | Regional |
| P2_ca_cs | Sol y vida (Asociacion cardiaca) | Spain | General heart disease | Regional |
| P2_ca_cs | ASPACOAR (Asociación de pacientes coronarios de Arahal) | Spain | General heart disease | Regional |
| P2_ca_cs | ASPACOLAS (Asociación de pacientes coronarios de Las Cabezas) | Spain | General heart disease | Regional |
| P1 | Asociación Española Contra la Muerte Súbita (Spanish Association against sudden death) | Spain | Sudden Cardiac Death | National |
| PS | Sociedad Espanola de Cardiologia (The Spanish Society of Cardiology)  represented by Prof. Merino | Spain | General Heart Disease | National |
| P3 | AGP (Alianza General de Pacientes) | Spain | General patient organization | National |
| P3 | FEP (Foro Español de Pacientes) | Spain | General patient organization | National |

**Social Media Groups**

| **Social Media Group** | **Country** | **Platform** | **Member Count** |
| --- | --- | --- | --- |
| defibrillator_icd_deutschland | Germany | Instagram | 721 |
| ICD / Implantierbarer Cardioverter Defibrillator | Germany | Facebook | 1792 |
| Herzkrank - Herzschrittmacher & ICD Implantiert | Germany | Facebook | 1742 |
| Personen mit Herzschrittmacher oder Defibrillator | Germany | Facebook | 2203 |
| Leben mit einem Herzschrittmacher | Germany | Facebook | 1026 |
| Herzinsuffizienz Patienten | Germany | Facebook | 3173 |
| SelbstHilfeGruppe für Herzerkrankungen | Germany | Facebook | 2247 |
| Living with a Medtronic Pacemaker, ICD, EV-ICD or CRT Device | Global | Facebook | 5937 |
| Pacemaker Club | Global | Facebook | 6200 |
| PACEMAKER // ICD AWARENESS (OPEN GROUP) | Global | Facebook | 9296 |
| Pacemaker and ICD Support Group | Global | Facebook | 9175 |
| Living with an Implantable Cardioverter Defibrillator ICD (Support Group) | Global | Facebook | 20239 |
| Sudden Cardiac Arrest Survivors | Global | Facebook | 7418 |
| Living with the S-ICD / EV-ICD | Global | Facebook | 3905 |
| CARDIAC ATHLETES (Pioneers of Sports Cardiology) | Global | Facebook | 6755 |
| Healthy Hearties ❤️ Cardiac Rehab | Global | Facebook | 3620 |
| ICD INTERNATIONAL SUPPORT. Uniting Those With Implanted Defibrillators | Global | Facebook | 855 |
| Surgery: Open Heart | Global | Facebook | 25417 |
| Heart Attack Survivors, getting heart healthy | Global | Facebook | 5092 |
| Defibrillator, Pacemaker And ICD Implant Support Group | Global | Facebook | 6502 |
| Heart Failure Support Group | Global | Facebook | 17911 |

All contacted organizations and patient groups that declined support were removed from the lists.
