# Supplementary material for: Patients’ Perspectives on Applications of AI and Personalized Medicine in Life-Threatening Heart Disease: European Cross-Sectional Patient Questionnaire Study
Source: J Med Internet Res. 2026 Aug 25;28:e96968. doi: 10.2196/96968 (PMC13505601; doi:10.2196/96968)
Supplement: Supplementary file 3 [file jmir-v28-e96968-s004.docx]

## Informed Consent and Questionnaire including Infographics

For this paper, we only include the version of the UK questionnaire, videos and images. This has two main reasons: The iterative construction of the instrument initially started in British English and after German translation and testing, it was translated and tested in the other languages. The first pre-launch and the first launch of the survey have also been in the UK. All other translations of questionnaires, videos and images can be provided by request to the corresponding author.

***Survey Title****: Patients’ Perspectives on Applications of Artificial Intelligence, Personalised Medicine and Non-inferiority Trials in Heart-Related Diseases*

##### 1) Introduction

Welcome to our survey!

Thank you for taking time out of your day to help us understand patient perspectives on artificial intelligence, personalised medicine, and non-inferiority trials. Don't worry if these concepts seem complex right now. More detailed information about the survey will be shared with you in the following sections. Your input is crucial in helping us understand how patients like yourself think about these topics.

How does participation impact you?

There are no risks associated with participating in this study. Please note that this initial section concerns your agreement to participate in the survey and how we handle your personal data. It outlines the data protection and privacy aspects of the survey. The survey takes approximately 25-30 minutes to complete. Keep in mind that you can interrupt the survey at any time and continue later.

Data Protection

Your participation is voluntary, and you may withdraw at any time without consequences. The survey ensures confidentiality, collecting no direct identifiers. Data will be securely stored, anonymised, and used solely for research. Results may be published while maintaining anonymity. By participating, you consent to data processing per General Data Protection Regulations (GDPR). Please review the full Data Consent Form below before proceeding.

Thank you for participating and sharing your valuable insights.

##### 2) Data protection Consent Form

I understand that my participation is **voluntary**, and I have the **right to withdraw** at any time without providing a reason. Withdrawing from the survey will not have any negative consequences for me.

I acknowledge that the survey will be conducted in a way that ensures confidentiality. While no direct personal identifiers (such as names or contact details) will be collected, some sociodemographic data may be gathered. Any data that could indirectly identify participants will be processed and stored securely and anonymised before analysis.

I understand that the information provided will be used solely for **research purposes** and will not be shared with any third parties. The results of this survey may be used for academic or scientific publications, but my identity will remain **confidential**.

Your data will be securely stored and pseudonymised during analysis to ensure confidentiality. All personal data will be deleted no later than three months after the research findings are finalised. In accordance with GDPR regulations, anonymised data may be retained for scientific research. Participants have the right to access, rectify, delete, restrict processing, object to processing, and request data portability, as per Articles 15–21 GDPR. If you wish to exercise these rights, please contact the research team at any time.

Processing of the gathered data during this study will be lawful and remain confidential within the limits established by the EU General Data Protection Regulation (GDPR) if you give consent to the processing of your data for the purpose described above in accordance with Article 6(1)(a) GDPR. Health-related data is processed based on Article 9(2)(a) GDPR.

For general inquiries about the study, please contact the research team. For concerns about data protection rights or complaints, you may directly contact the Data Protection Officer or the Bavarian Data Protection Agency: Data Protection Officer of the University of Bayreuth, Jonas Holoubek, [datenschutz@uni-bayreuth.de](mailto:datenschutz@uni-bayreuth.de); The Bavarian Data Protection Agency, Promenade 27, 91522 Ansbach, Germany.

**How to contact us:** [PROFID-Project@uni-bayreuth.de](mailto:PROFID-Project@uni-bayreuth.de)
Please do not hesitate to reach out to us in your native language or English.

**Responsible Scientists (University of Bayreuth, Germany)**
Project Leaders Survey Study: Professor Dr. mult. Eckhard Nagel, Professor Dr. Michael Lauerer
Operative Scientific Lead: Georg Lindinger
Researchers: Nicolas Schiermeier, Mona Khattab, Maximilian Dichtl

*Agree Button: I consent to participate in the study and agree to the processing of my personal data in accordance with the information provided herein.*

##### 3) Description of research and role of participants

What Is the Survey About?

This questionnaire is part of the PROFID project. The project aims to improve how patients are selected for a cardiac device called an implantable cardioverter defibrillator (ICD) after suffering a heart attack to reduce the risk of sudden cardiac death. It investigates whether patients require a defibrillator and medication or if 'solely' taking the right medications could suffice for some patients, potentially leading to fewer side effects and improved quality of life. To learn more about the project, please click this link: <https://profid-project.eu/>

The questionnaire addresses questions that arise from the PROFID project and covers three topics: (1) personalised medicine (PM), (2) artificial intelligence (AI) in healthcare, and (3) non-inferiority trials. Your answers will help us learn more about the perspectives of patients and help to improve healthcare decisions and treatments. Don't worry if you are not familiar with the topics of the survey yet. Explanations in the form of explanatory videos or alternatively infographics are provided before each question section.

Your answers are important and will be kept confidential. This survey is anonymous. There are no right or wrong answers –just tell us what you think!

Your feedback will help us to integrate the perspectives of patients and their preferences in research and recent developments in healthcare across your country and Europe.

Your feedback matters! Thank you for taking the time to share your thoughts with us!

##### 4) Personal Information

First, we’d like to know some information about you. We ensure all your responses are handled privately.

i) **Q1:** Which gender do you identify with?
   Female / Male / Other / Prefer not to say
ii) **Q 2:** What is your age? (in years)
    *Open*
iii) **Q 3**: What is your current country of residency?

*Country where the survey is conducted / Other*

iv) **Q 4:** Have you ever had a heart attack?
    Yes / No / Prefer not to say
v) **Q 5:** Have you been diagnosed with heart failure?
   Yes / No / Prefer not to say
vi) **Q 6:** Have you been resuscitated after your heart stopped?

Yes / No / Prefer not to say

vii) **Q 7:** Do you have an implantable defibrillator (ICD)?
     Yes, I have an ICD / Yes, I have a S-ICD / Yes, I have an CRT-D / Yes, I have an ICD or CRT-D, but I'm unsure about the type / No / Prefer not to say
     *Includes explanatory Info Box: ICD = Transvenous Implantable Cardioverter Defibrillator S-ICD = Subcutaneous Implantable Cardioverter Defibrillator CRT-D = Cardiac Resynchronisation Therapy Defibrillator*
viii) **Q 8:** How long do you have an ICD implanted? (in years)
      Less than 1 year / More than one year, namely: *Open* / Prefer not to say
ix) **Q 9:** What is your highest educational qualification?
    No formal qualifications / GCSEs (or equivalent) / A-Levels (or equivalent) / Vocational qualification (e.g., BTEC, NVQ) / Undergraduate degree (e.g., Bachelor's) / Postgraduate degree (e.g., Master's, PhD) / Prefer not to say / Other, please specify: *open*
x) **Q 10:** What's your current employment status?
   Student / Employed / Not employed / Retired / Prefer not to say
xi) **Q 11: Subjective social status (SSS)**
    *“At the top of the ladder are the people who are the best off, those who have the most money, most education, and best jobs. At the bottom are the people who are the worst off, those who have the least money, least education, worst jobs, or no job. Please place an ‘X’ on the rung that best represents where you think you stand on the ladder.”*
    Scale: Lowest / Highest (10 steps / without numbers)

**Includes Graphic: Mac Arthur Scale: Ladder**

##### 5) Patients’ Perspectives on Technology

Answers: Strongly Disagree / Disagree / Neutral / Agree / Strongly Agree/ Prefer not to say

i. New technologies contribute to a better quality of life.
ii. Technology gives me more freedom of mobility.
iii. Technology gives people more control over their daily lives.
iv. Technology makes me more productive in my personal life.
v. Other people come to me for advice on new technologies.
vi. In general, I am among the first in my circle of friends to acquire new technology when it appears.
vii. I can usually figure out new high-tech products and services without help from others.
viii. I keep up with the latest technological developments in my areas of interest.
ix. When I get technical support from a provider of a high-tech product or service, I sometimes feel as if I am being taken advantage of by someone who knows more than I do.
x. Technical support lines are not helpful because they don’t explain things in terms I understand.
xi. Sometimes, I think that technology systems are not designed for use by ordinary people.
xii. There is no such thing as a manual for a high-tech product or service that’s written in plain language.
xiii. People are too dependent on technology to do things for them.
xiv. Too much technology distracts people to a point that is harmful.
xv. Technology lowers the quality of relationships by reducing personal interaction.
xvi. I do not feel confident doing business with a place that can only be reached online.

##### 6) Patients’ Perspectives on Medical Research

i. I want to be part of medical research to improve medical progress.
ii. I want to be part of medical research to improve benefits for all patients.
iii. I want to be part of medical research to improve the healthcare system.
iv. I want to be part of medical research to get the best possible medical outcome for myself.

##### 7) Patients’ Perspectives on Personalised Medicine

In the following section, we would like to know your perspectives on the use of personalised medicine in making treatment decisions.

*The graphics are accompanied by a short explanatory infographics video.*

To what extent do you agree or disagree with the following statements?

i. I believe personalised risk prediction models improve the accuracy of medical diagnoses.
ii. I believe personalised risk prediction models improve the accuracy of medical treatment decisions.
iii. I want to know if a personalised risk prediction model is involved in my treatment decisions.
iv. I want to be informed, how exactly the personalised risk prediction model is used to influence my treatment decisions.
v. I want to take part in the decision of whether to implant an ICD (S-ICD/ CRT-D).
vi. I want to take part in the decision of whether to implant an ICD (S-ICD/ CRT-D), if a personalised risk prediction model is used.
vii. Personalised risk prediction models should support treatment decisions of healthcare providers.
viii. Health care providers should use personalised risk prediction models to support the decision whether to implant an ICD (S-ICD/CRT-D).
ix. Health care providers should have to base their decision whether to implant an ICD (S-ICD/CRT-D) on risk prediction models.
x. Risk prediction models should make the decision whether to implant an ICD (S-ICD/CRT-D).

Answers: Strongly Disagree / Disagree / Neutral / Agree / Strongly Agree/ Prefer not to say

- Which opportunities do you think personalised risk prediction models offer to heart patients?

*Open*

- Which concerns do you have about the use of personalised risk prediction models for heart patients?

*Open*

##### 8) Patients’ Perspectives on Artificial Intelligence

In the following section, we would like to know your views on artificial intelligence in healthcare.

Answers: Strongly Disagree / Disagree / Neutral / Agree / Strongly Agree/ Prefer not to say

i. I am interested in Artificial Intelligence (AI).
ii. In my view, I have a good understanding of AI in general.
iii. How often do you use applications based on AI technology in your daily life?

Never / rarely / sometimes / often / always / Prefer not to say

By Artificial Intelligence (AI) in healthcare, we refer to the following:

*The graphics are accompanied by a short explanatory infographics video.*

To what extent do you agree or disagree with the following statements?

i. I believe AI can improve the accuracy of medical diagnoses.
ii. I believe AI can improve the accuracy of medical treatment decisions.
iii. I want to know if AI is involved in my treatment decisions.
iv. I want to be informed about how exactly AI is used in making my treatment decisions.
v. AI should be used to support treatment decisions.
vi. Recommendations made by AI should complement the role of healthcare providers in treatment decisions.
vii. I think healthcare providers should bear responsibility for the consequences of medical decisions supported by AI.
viii. I am comfortable with AI making medical decisions.
ix. I am concerned AI will replace human expertise in the medical field.
x. I want to take part in the decision of whether to implant an ICD (S-ICD/ CRT-D), if AI is used.
xi. Health care providers should use AI to support the decision whether to implant an ICD (S-ICD/CRT-D).
xii. Health care providers should have to base their decision whether to implant an ICD (S-ICD/CRT-D) on AI.
xiii. AI should make the decision whether to implant an ICD (S-ICD/CRT-D).

- *Which opportunities do you think AI offers to improve healthcare for heart patients?*

*Open*

- *Which concerns do you have about the use of AI in healthcare for heart patients?*

*Open*

In the previous sections we asked you questions about AI and personalised risk prediction models. AI technology can be used in personalised risk prediction models in medicine.

- The use of AI technology influences my opinion concerning personalised risk prediction models.
  Answers: Strongly Disagree / Disagree / Neutral / Agree / Strongly Agree/ Prefer not to say
- How does the use of AI influence your opinion?
  Answers: Strongly Negative / Negative / slightly Negative /Neutral / slightly positive / positive / strongly positive

##### 9) Patients’ Perspectives on Non-inferiority Trials

In the following section, we would like to know your perspectives on ethical considerations in Non-inferiority Trials.

*The graphics are accompanied by a short explanatory infographics video.*

To what extent do you agree or disagree with the following statements?

i. I want to be informed about the purpose of a non-inferiority trial, even if it's complex.
ii. I would feel comfortable about being randomised to one of the treatment groups in a non-inferiority trial.
iii. I would be willing to participate in a non-inferiority trial even if there is a small potentially higher risk of death, but there are other important advantages (e.g. reduced side effects, improved quality of life).
iv. I want to take part in a non-inferiority trial to improve the healthcare system in general by reducing costs.
v. I want to take part in a non-inferiority trial to improve the healthcare system in general by improving medical treatment options for others.
vi. Patients should have a say in deciding, if the benefits, like reduced side effects, outweigh any potential decrease in treatment effectiveness (e.g. lower survival rate).

- What are your feelings concerning taking part in a non-inferiority trial?

*Open*

##### 10) Closing

Thank you for participating in this survey! Your feedback will help us to integrate the perspectives of patients and their preferences in research and recent developments in healthcare in your country and in Europe.

Comments: open box

For any questions, please contact us: [PROFID-Project@uni-bayreuth.de](mailto:PROFID-Project@uni-bayreuth.de)
Please do not hesitate to reach out to us in your native language or English.

**Comments**:
