# Supplementary material for: Patients’ Perspectives on Applications of AI and Personalized Medicine in Life-Threatening Heart Disease: European Cross-Sectional Patient Questionnaire Study
Source: J Med Internet Res. 2026 Aug 25;28:e96968. doi: 10.2196/96968 (PMC13505601; doi:10.2196/96968)
Supplement: Supplementary file 4 [file jmir-v28-e96968-s005.docx]

## Statistical Calculations and Quality Assurance Measures

Table 1: Subsample characteristics (n=354)

| Variable | GER, n (%) | NET, n (%) | UK, n (%) | Other**^a^**, n (%) | Total, N (%) |
| --- | --- | --- | --- | --- | --- |
| **Population** | 177 (-) | 58 (-) | 104 (-) | 15 | 354 (-) |
| **Gender** |  |  |  |  |  |
| Male | 111 (62.7) | 27 (46.6) | 45 (43.3) | 7 (46.7) | 190 (53.7) |
| Female | 66 (37.3) | 31 (53.4) | 59 (56.7) | 8 (53.3) | 164 (46.3) |
| **Age** |  |  |  |  |  |
| 18-44 | 12 (6.8) | 7 (12.1) | 9 (8.7) | 4 (26.7) | 31 (8.8) |
| 45-54 | 30 (16.9) | 8 (13.8) | 15 (14.4) | 4 (26.7) | 57 (16.1) |
| 55-64 | 48 (27.1) | 22 (37.9) | 40 (38.5) | 2 (13.3) | 113 (31.9) |
| 65-74 | 51 (28.8) | 17 (29.3) | 31 (29.8) | 3 (20) | 103 (29.1) |
| 75+ | 36 (20.3) | 4 (6.9) | 9 (8.7) | 2 (13.3) | 50 (14.1) |
| **Cardiac History^b^** |  |  |  |  |  |
| Myocardial infarction | 79 (44.6) | 19 (32.8) | 25 (24) | 8 (53.3) | 131 (37) |
| Heart failure | 116 (65.5) | 47 (81) | 71 (68.3) | 14 (6.7) | 248 (70.1) |
| Cardiac arrest | 56 (31.6) | 29 (50) | 33 (31.7) | 2 (13.3) | 120 (33.9) |
| **ICD implanted** |  |  |  |  |  |
| ICD/S-ICD | 58 (32.8) | 51 (49) | 51 (49) | 3 (20) | 150 (42.4) |
| CRT-D | 10 (5.6) | 5 (4.8) | 5 (4.8) | 1 (6.7) | 26 (7.3) |
| Unsure about device type | 7 (4) | - | - | 1 (6.7) | 9 (2.5) |
| No | 102 (57.6) | 48 (46.2) | 48 (46.2) | 10 (66.7) | 169 (47.7) |
| **Educational attainment** |  |  |  |  |  |
| GCSEs (or equivalent) | 27 (15.3) | 11 (19) | 17 (16.3) | 4 (26.7) | 59 (16.7) |
| A-Levels (or equivalent) | 26 (14.7) | 1 (1.7) | 4 (3.8) | 1 (6.7) | 32 (9) |
| Vocational qualification | 39 (22) | 27 (46.6) | 19 (18.3) | 2 (13.3) | 87 (24.6) |
| Undergraduate degree | 21 (11.9) | 11 (19) | 39 (37.5) | 5 (33.3) | 76 (21.5) |
| Postgraduate degree | 61 (34.5) | 7 (12.1) | 24 (23.3) | 3 (20) | 95 (26.8) |
| **Employment status** |  |  |  |  |  |
| Employed or self employed | 67 (37.9) | 28 (48.3) | 31 (29.8) | 7 (46.7) | 133 (37.6) |
| Not employed | 2 (1.1) | 8 (13.8) | 14 (13.5) | 1 (6.7) | 25 (7.1) |
| Student or in training | 1 (0.6) | - | 1 (1) | - | 2 (0.6) |
| Retired | 103 (58.2) | 19 (32.8) | 56 (53.8) | 5 (33.3) | 183 (51.7) |
| Unable to work | 2 (1.1) | 2 (3.4) | 1(1) | 2 (13.3) | 7 (2) |
| Prefer not to say | 2 (1.1) | 1 (1.7) | 1 (1) | - | 4 (1.1) |
| **Subjective social status** |  |  |  |  |  |
| Low (1-4) | 22 (12.4) | 6 (10.3) | 15 (14.4) | 1 (6.7) | 44 (12.4) |
| Medium (5-7) | 128 (72.3) | 34 (58.6) | 66 (63.5) | 13 (86.7) | 241 (68.1) |
| High (8-10) | 27 (15.3) | 18 (31) | 23 (22.1) | 1 (6.7) | 69 (19.5) |
| ^a^Other countries: Austria (n=4); Belgium (n=3); Spain (n=8).  ^b^Multiple answers possible. | | | | | |

**Table 2: Correlation matrix of the EFA**

| Factor | 1^a^ | 2^b^ | 3^c^ | 4^d^ | 5^e^ | 6^f^ |
| --- | --- | --- | --- | --- | --- | --- |
| 1^a^ | 1.000 | .124 | .152 | .375 | -.446 | -.230 |
| 2^b^ | .124 | 1.000 | -.111 | .076 | -.287 | -.488 |
| 3^c^ | .152 | -.111 | 1.000 | -.032 | -.213 | .042 |
| 4^d^ | .375 | .076 | -.032 | 1.000 | -.201 | -.155 |
| 5^e^ | -.446 | -.287 | -.213 | -.201 | 1.000 | .326 |
| 6^f^ | -.230 | -.488 | .042 | -.155 | .326 | 1.000 |
| Extraction method: principal axis factoring. Rotation method: direct oblimin with Kaiser normalization  ^a^Perceived Benefits and Support for Usage of AI in MDM  ^b^Transparency Expectations in Algorithmic Decision-Making  ^c^Support for Delegation of Decisions to Algorithms  ^d^Self-Reported AI Literacy  ^e^Perceived Benefits and Support for Usage of PRP in MDM  ^f^Preference for Shared Decision-Making | | | | | | |

**Table 3: Means and SDs of the items included in the EFA by country**

| Factors and Items | GER Mean^a^ (SD) | NET Mean^a^ (SD) | | UK Mean^a^ (SD) | | | Other Mean^a^ (SD) | | |  |
| --- | --- | --- | --- | --- | --- | --- | --- | --- | --- | --- |
| **Perceived Benefits and Support for Usage of PRP in MDM** | | | | | |  | | |  |  |
| I believe personalised risk prediction models improve the accuracy of medical diagnoses. | 4.32 (0.66) | | 4.05 (0.69) | | 4.05 (0.79) | | | 4.09 (1.15) | | |
| I believe personalised risk prediction models improve the accuracy of medical treatment decisions. | 4.27 (0.68) | | 4.09 (0.65) | | 4.08 (0.68) | | | 4.23 (1.07) | | |
| Health care providers should use personalised risk prediction models to support the decision whether to implant an ICD (S-ICD/CRT-D). | 4.33 (0.71) | | 4.09 (0.76) | | 4.08 (0.75) | | | 4.05 (0.87) | | |
| Personalised risk prediction models should support treatment decisions of healthcare providers. | 4.41 (0.62) | | 4.23 (0.7) | | 4.20 (0.78) | | | 4.05 (0.79) | | |
| **Perceived Benefits and Support for Usage of AI in MDM** | | | | | |  | | |  |  |
| I believe AI can improve the accuracy of medical diagnoses. | 4.14 (0.79) | | 3.84 (0.72) | | 3.76 (0.95) | | | 3.81 (0.83) | | |
| I believe AI can improve the accuracy of medical treatment decisions. | 4.02 (0.82) | | 3.74 (0.72) | | 3.64 (0.94) | | | 4.13 (0.62) | | |
| AI should be used to support treatment decisions. | 4.14 (0.8) | | 3.79 (0.91) | | 3.66 (0.96) | | | 3.73 (0.7) | | |
| Health care providers should use AI to support the decision whether to implant an ICD (S-ICD/CRT-D). | 4.08 (0.76) | | 3.67 (0.89) | | 3.56 (0.95) | | | 3.6 (1.18) | | |
| Recommendations made by AI should complement the role of healthcare providers in treatment decisions. | 4.05 (0.78) | | 3.91 (0.78) | | 4.18 (0.91) | | | 3.88 (0.72) | | |
| **Transparency Expectations in Algorithmic Decision-Making** | | | | | | | | |  |  |
| I want to be informed about how exactly AI is used in making my treatment decisions. | 4.45 (0.73) | | 4.07 (0.88) | | 4.49 (0.64) | | | 4.31 (1.01) | | |
| I want to know if AI is involved in my treatment decisions. | 4.53 (0.61) | | 4.28 (0.72) | | 4.49 (0.64) | | | 4.06 (1.06) | | |
| I want to be informed how exactly the personalised risk prediction model is used to influence my treatment decisions. | 4.52 (0.61) | | 4.15 (0.86) | | 4.51 (0.55) | | | 4 (1.2) | | |
| I want to know if a personalised risk prediction model is involved in my treatment decisions. | 4.49 (0.59) | | 4.29 (0.7) | | 4.47 (0.54) | | | 4.05 (1.13) | | |
| **Support for Delegation of Decisions to Algorithms** |  | |  | |  | | |  | | |
| Risk prediction models should make the decision whether to implant an ICD (S-ICD/CRT-D). | 2.67 (1.22) | | 3.55 (1.08) | | 2.71 (1.09) | | | 3.38 (1.36) | | |
| Health care providers should have to base their decision whether to implant an ICD (S-ICD/CRT-D) on risk prediction models. | 3.49 (1.1) | | 3.41 (1.08) | | 3.26 (1.04) | | | 3.82 (0.91) | | |
| AI should make the decision whether to implant an ICD (S-ICD/CRT-D). | 1.66 (0.81) | | 1.98 (0.69) | | 1.91 (0.86) | | | 1.8 (0.86) | | |
| Health care providers should have to base their decision whether to implant an ICD (S-ICD/CRT-D) on AI. | 2.72 (0.97) | | 2.29 (0.86) | | 2.44 (1.04) | | | 2.73 (1.16) | | |
| **Self-Reported AI Literacy** |  | |  | |  | | |  | | |
| In my view. I have a good understanding of AI in general. | 2.78 (1) | | 3.38 (0.88) | | 3.16 (1.09) | | | 3.11 (0.94) | | |
| How often do you use applications based on AI technology in your daily life? | 2.68 (1.06) | | 2.71 (1.09) | | 2.7 (1.02) | | | 2.58 (1.17) | | |
| I am interested in artificial intelligence (AI). | 3.94 (0.93) | | 3.71 (1.04) | | 3.72 (1.02) | | | 3.53 (1.12) | | |
| **Preference for Shared Decision-Making** |  | |  | |  | | |  | | |
| I want to take part in the decision of whether to implant an ICD (S-ICD/ CRT-D). | 4.62 (0.69) | | 4.24 (0.81) | | 4.47 (0.78) | | | 3.81 (1.29) | | |
| I want to take part in the decision of whether to implant an ICD (S-ICD/ CRT-D), if a personalised risk prediction model is used. | 4.57 (0.69) | | 4.18 (0.82) | | 4.52 (0.6) | | | 4.1 (1.04) | | |
| I want to take part in the decision of whether to implant an ICD (S-ICD/ CRT-D), if AI is used. | 4.57 (0.56) | | 4.4 (0.67) | | 4.48 (0.68) | | | 4.07 (0.92) | | |
| ^a^Sample sizes for means/SDs differ across scales due to missing responses. GER (n=175-191); NET (n=57-66); UK (n=105-114); Other (AUT. n=4-6; BEL. n=3-4; SPA. n=7-12). | | | | | | | | |  |  |

#### Internal consistency of the identified factors

**Table 4: Reliability statistics of the factor “Perceived Benefits and Support for Usage of PRP in MDM”**

| Cronbach Alpha | Number of Items |
| --- | --- |
| .854 | 4 |

**Table 5: Item-Scale Statistics of the factor “Perceived Benefits and Support for Usage of PRP in MDM”**

| Item | Scale mean when the item is omitted | Scale variance when an item is omitted | Corrected item-scale correlation | Cronbach alpha when an item is omitted |
| --- | --- | --- | --- | --- |
| I believe personalised risk prediction models improve the accuracy of medical diagnoses. | 12.68 | 3.309 | .731 | .799 |
| I believe personalised risk prediction models improve the accuracy of medical treatment decisions. | 12.68 | 3.433 | .737 | .798 |
| Personalised risk prediction models should support treatment decisions of healthcare providers. | 12.56 | 3.671 | .635 | .839 |
| Health care providers should use personalised risk prediction models to support the decision whether to implant an ICD (S-ICD/CRT-D). | 12.66 | 3.403 | .684 | .820 |

**Table 6: Reliability statistics of the factor “Perceived Benefits and Support for Usage of AI in MDM”**

| Cronbach Alpha | Number of Items |
| --- | --- |
| .895 | 5 |

**Table 7: Item-Scale Statistics of the factor “Perceived Benefits and Support for Usage of AI in MDM”**

| Item | Scale mean when the item is omitted | Scale variance when an item is omitted | Corrected item-scale correlation | Cronbach alpha when an item is omitted |
| --- | --- | --- | --- | --- |
| I believe AI can improve the accuracy of medical diagnoses. | 15.71 | 8.271 | .804 | .859 |
| I believe AI can improve the accuracy of medical treatment decisions. | 15.80 | 8.328 | .781 | .864 |
| AI should be used to support treatment decisions. | 15.75 | 8.129 | .766 | .867 |
| Recommendations made by AI should complement the role of healthcare providers in treatment decisions. | 15.61 | 8.892 | .674 | .887 |
| Health care providers should use AI to support the decision whether to implant an ICD (S-ICD/CRT-D). | 15.83 | 8.446 | .691 | .884 |

**Table 8: Reliability statistics of the factor “Transparency Expectations in Algorithmic Decision-Making”**

| Cronbach Alpha | Number of Items |
| --- | --- |
| .841 | 4 |

**Table 9: Item-Scale Statistics of the factor “Transparency Expectations in Algorithmic Decision-Making”**

| Item | Scale mean when the item is omitted | Scale variance when an item is omitted | Corrected item-scale correlation | Cronbach alpha when an item is omitted |
| --- | --- | --- | --- | --- |
| I want to know if a personalised risk prediction model is involved in my treatment decisions. | 13.30 | 3.144 | .664 | .806 |
| I want to be informed how exactly the personalised risk prediction model is used to influence my treatment decisions. | 13.29 | 2.928 | .691 | .792 |
| I want to know if AI is involved in my treatment decisions. | 13.29 | 2.888 | .713 | .782 |
| I want to be informed about how exactly AI is used in making my treatment decisions. | 13.35 | 2.739 | .649 | .816 |

**Table 10: Reliability statistics of the factor “Support for Delegation of Decisions to Algorithms”**

| Cronbach Alpha | Number of Items |
| --- | --- |
| .758 | 4 |

**Table 11: Item-Scale Statistics of the factor “Support for Delegation of Decisions to Algorithms”**

| Item | Scale mean when the item is omitted | Scale variance when an item is omitted | Corrected item-scale correlation | Cronbach alpha when an item is omitted |
| --- | --- | --- | --- | --- |
| Health care providers should have to base their decision whether to implant an ICD (S-ICD/CRT-D) on risk prediction models. | 7.22 | 5.480 | .665 | .638 |
| Health care providers should have to base their decision whether to implant an ICD (S-ICD/CRT-D) on AI. | 8.06 | 6.498 | .501 | .730 |
| Risk prediction models should make the decision whether to implant an ICD (S-ICD/CRT-D). | 7.78 | 5.183 | .606 | .678 |
| AI should make the decision whether to implant an ICD (S-ICD/CRT-D). | 8.83 | 7.236 | .484 | .742 |

**Table 12: Reliability statistics of the factor “Self-Reported AI Literacy”**

| Cronbach Alpha | Number of Items |
| --- | --- |
| .797 | 3 |

**Table 13: Item-Scale Statistics of the factor “Transparency Expectations in Algorithmic Decision-Making”.**

| Item | Scale mean when the item is omitted | Scale variance when an item is omitted | Corrected item-scale correlation | Cronbach alpha when an item is omitted |
| --- | --- | --- | --- | --- |
| I am interested in artificial intelligence (AI). | 5.71 | 3.456 | .617 | .749 |
| I have a good understanding of AI in general. | 6.53 | 3.306 | .605 | .762 |
| How often do you use applications based on AI technology in your daily life? | 6.84 | 2.957 | .706 | .652 |

**Table 14: Reliability statistics of the factor “Preference for Shared Decision-Making”**

| Cronbach Alpha | Number of Items |
| --- | --- |
| .843 | 3 |

**Table 15: Item-Scale Statistics of the factor “Preference for Shared Decision-Making”**

| Item | Scale mean when the item is omitted | Scale variance when an item is omitted | Corrected item-scale correlation | Cronbach alpha when an item is omitted |
| --- | --- | --- | --- | --- |
| I want to take part in the decision of whether to implant an ICD (S-ICD/ CRT-D). | 8.99 | 1.390 | .763 | .730 |
| I want to take part in the decision of whether to implant an ICD (S-ICD/ CRT-D), if a personalised risk prediction model is used. | 8.99 | 1.595 | .741 | .750 |
| I want to take part in the decision of whether to implant an ICD (S-ICD/ CRT-D), if AI is used. | 9.00 | 1.840 | .636 | .848 |

**Table 16: Correlations among scales (Pearson r and two-tailed P value)**

| Factor | | Benefits/ Support PRP | Benefits/ Support  AI | Self-reported AI Literacy | Delegation Support to Algorithms | Transparency Expectations | Preference for SDM |
| --- | --- | --- | --- | --- | --- | --- | --- |
| **Benefits/Support PRP** | |  |  |  |  |  |  |
|  | *r* | 1 | .549^a^ | .298^a^ | .340^a^ | .349^a^ | .286^a^ |
|  | *P* |  | <.001 | <.001 | <.001 | <.001 | <.001 |
|  | n | 392 | 354 | 372 | 354 | 354 | 351 |
| **Benefits/Support AI** | |  |  |  |  |  |  |
|  | *r* | .549^a^ | 1 | .445^a^ | .213^a^ | .206^a^ | .227^a^ |
|  | *P* | <.001 |  | <.001 | <.001 | <.001 | <.001 |
|  | N | 354 | 355 | 349 | 355 | 354 | 352 |
| **Self-reported AI Literacy** | |  |  |  |  |  |  |
|  | *r* | .298^a^ | .445^a^ | 1 | .037 | .137^b^ | .167^a^ |
|  | *P* | <.001 | <.001 |  | .486 | .010 | .002 |
|  | N | 372 | 349 | 374 | 349 | 349 | 346 |
| **Delegation Support to Algorithms** | |  |  |  |  |  |  |
|  | *r* | .340^a^ | .213^a^ | .037 | 1 | -.062 | -.040 |
|  | *P* | <.001 | <.001 | .486 |  | .246 | .452 |
|  | N | 354 | 355 | 349 | 355 | 354 | 352 |
| **Transparency Expectations** | |  |  |  |  |  |  |
|  | *r* | .349^a^ | .206^a^ | .137^b^ | -.062 | 1 | .498^a^ |
|  | *P* | <.001 | <.001 | .010 | .246 |  | <.001 |
|  | N | 354 | 354 | 349 | 354 | 355 | 352 |
| **Preference for SDM** | |  |  |  |  |  |  |
|  | *r* | .286^a^ | .227^a^ | .167^a^ | -.040 | .498^a^ | 1 |
|  | *P* | <.001 | <.001 | .002 | .452 | <.001 |  |
|  | N | 351 | 352 | 346 | 352 | 352 | 352 |
| ^a^*P* < .01 (2-tailed).  ^b^*P* < .05 (2-tailed). | | | | | | | |

**Table 17: Multiple linear regression adjusting the independent-samples t-test comparing AI benefits between men and women (n=344).**

|  |  | |  |  | 95% CI | |
| --- | --- | --- | --- | --- | --- | --- |
| Coefficient | B | rSE^a^ | T | *P* | lower | upper |
| (constant) | 0.934 | 0.326 | 2.862 | .004 | 0.292 | 1.575 |
| Gender^b^ | -.125 | .077 | -1.638 | .102 | -.276 | .025 |
| Age | .009 | .003 | 2.761 | .006 | .003 | .015 |
| Educational attainment | .046 | .025 | 1.803 | .072 | -.004 | .096 |
| Self-Reported AI Literacy | .370 | .049 | 7.505 | <.001 | .273 | .467 |

^a^ HC3 Method.

^b^ Gender coded as men=0 and women=1.

Note: The overall model was statistically significant, F(4,339)=27.68, P<.001, explaining 24.6% of the variance in perceived AI benefits (R²=.246; adjusted R²=.237). The modified Breusch-Pagan test indicated significant heteroscedasticity, χ²(1)=26.33, P<.001. Therefore, HC3 robust standard errors were applied.

**Table 18: Country-adjusted multiple linear regression associated with perceived support and benefits of PRP in MDM with Germany as baseline (n=333)**

|  |  | |  |  | 95% CI | |
| --- | --- | --- | --- | --- | --- | --- |
| Coefficient | B | rSE^a^ | T | *P* | lower | upper |
| (constant) | .949 | .362 | 2.622 | .009 | .237 | 1.660 |
| TRI Score | .110 | .067 | 1.651 | .100 | -.021 | .242 |
| Self-Reported AI Literacy | .117 | .049 | 2.398 | .017 | .021 | .212 |
| Delegation of Decisions to Algorithms | .252 | .047 | 5.351 | <.001 | .159 | .344 |
| Transparency in Algorithmic Decision-Making | .261 | .063 | 4.163 | <.001 | .137 | .384 |
| Shared Decision-Making | .109 | .064 | 1.718 | .087 | -.016 | .235 |
| Educational attainment | .037 | .026 | 1.457 | .146 | -.013 | .088 |
| Gender^b^ | .044 | .061 | .720 | .472 | -.077 | .165 |
| Age | .003 | .003 | 1.255 | .210 | -.002 | .008 |
| Country Netherlands | -.154 | .069 | -2.244 | .026 | -.289 | -.019 |
| Country UK | -.227 | .068 | -3.359 | <.001 | -.361 | -.094 |

^a^ HC3 Method.

^b^ Gender coded as men=0 and women=1.

Note: The overall model was statistically significant, F(8,331)=18.93, P<.001, explaining 31.4% of the variance in perceived PRP benefits (R²=.314; adjusted R²=.297). The modified Breusch-Pagan test indicated significant heteroscedasticity, χ²(1)=8.14, P=.004. Therefore, HC3 robust standard errors were applied.

**Table 19: County-adjusted multiple linear regression associated with perceived support and benefits of AI in MDM with Germany as baseline (n=334)**

|  |  | |  |  | 95% CI | |
| --- | --- | --- | --- | --- | --- | --- |
| Coefficient | B | rSE^a^ | T | *P* | lower | upper |
| (constant) | .454 | .463 | .980 | .328 | -.457 | 1.365 |
| TRI Score | .246 | .083 | 2.953 | .003 | .082 | .410 |
| Self-Reported AI Literacy | .243 | .064 | 3.815 | <.001 | .118 | .368 |
| Delegation of Decisions to Algorithms | .171 | .041 | 4.189 | <.001 | .091 | .251 |
| Transparency in Algorithmic Decision-Making | .152 | .067 | 2.268 | .024 | .020 | .284 |
| Shared Decision-Making | .091 | .066 | 1.387 | .166 | -.038 | .221 |
| Educational attainment | .041 | .026 | 1.606 | .109 | -.009 | .092 |
| Gender^b^ | -.106 | .073 | -1.437 | .152 | -.250 | .039 |
| Age | .007 | .003 | 2.347 | .020 | .001 | .012 |
| Country Netherlands | -.286 | .083 | -3.431 | <.001 | -.450 | -.122 |
| Country UK | -.259 | .076 | -3.420 | <.001 | -.408 | -.110 |

^a^ HC3 Method.

^b^ Gender coded as men=0 and women=1.

Note: The overall model was statistically significant, F(8,332)=21.15, P<.001, explaining 33.8% of the variance in perceived AI benefits (R²=.338; adjusted R²=.322). The residuals showed some deviation from normality. Visual inspection as well as the modified Breusch-Pagan test indicated significant heteroscedasticity, χ²(1)=17.16, P<.001. Therefore, HC3 robust standard errors were applied.

**Table 20: Benjamin Hochberg adjustment for primary regression predictor coefficients at α=0.05**

| Model | Predictor | p | k | m | p(k)*m/k | BH adjusted p-value |
| --- | --- | --- | --- | --- | --- | --- |
| PRP-Benefits | Delegation of Decisions to Algorithms | 0.001^a^ | 1 | 16 | 0.016 | 0.004 |
| PRP-Benefits | Transparency in Algorithmic Decision-Making | 0.001^a^ | 2 | 16 | 0.008 | 0.004 |
| AI-Benefits | Self-Reported AI Literacy | 0.001^a^ | 3 | 16 | 0.005 | 0.004 |
| AI-Benefits | Delegation of Decisions to Algorithms | 0.001^a^ | 4 | 16 | 0.004 | 0.004 |
| AI-Benefits | TRI Score | 0.006 | 5 | 16 | 0.019 | 0.019 |
| AI-Benefits | Transparency in Algorithmic Decision-Making | 0.007 | 6 | 16 | 0.019 | 0.019 |
| AI-Benefits | Age | 0.030 | 7 | 16 | 0.069 | 0.069 |
| AI-Benefits | Educational attainment | 0.041 | 8 | 16 | 0.082 | 0.082 |
| AI-Benefits | Gender | 0.047 | 9 | 16 | 0.084 | 0.084 |
| PRP-Benefits | TRI Score | 0.059 | 10 | 16 | 0.094 | 0.086 |
| PRP-Benefits | Self-Reported AI Literacy | 0.059 | 11 | 16 | 0.086 | 0.086 |
| PRP-Benefits | Shared Decision-Making | 0.068 | 12 | 16 | 0.091 | 0.091 |
| PRP-Benefits | Educational attainment | 0.083 | 13 | 16 | 0.102 | 0.102 |
| AI-Benefits | Shared Decision-Making | 0.115 | 14 | 16 | 0.131 | 0.131 |
| PRP-Benefits | Age | 0.311 | 15 | 16 | 0.332 | 0.332 |
| PRP-Benefits | Gender | 0.805 | 16 | 16 | 0.805 | 0.805 |

^a^For FDR correction, P values reported by SPSS as P<.001 were conservatively entered as P=.001.

**Table 21: Descriptive statistics of EFA factor and TRI score means for Germany, the Netherlands and the United Kingdom.**

| Scale | Country | N | Mean | SD | SE | CI lower | CI upper | Minimum | Maximum |
| --- | --- | --- | --- | --- | --- | --- | --- | --- | --- |
| **Perceived Benefits and Support for Usage of PRP in MDM** | | | | | | | | | |
|  | Germany | 190 | 4.33 | 0.574 | 0.042 | 4.252 | 4.416 | 2.25 | 5 |
|  | Netherlands | 66 | 4.11 | 0.582 | 0.072 | 3.971 | 4.257 | 2.25 | 5 |
|  | United Kingdom | 114 | 4.1 | 0.609 | 0.057 | 3.99 | 4.216 | 2 | 5 |
|  | Total | 370 | 4.22 | 0.596 | 0.031 | 4.163 | 4.285 | 2 | 5 |
| **Perceived Benefits and Support for Usage of AI in MDM** | | | | | | | | | |
|  | Germany | 177 | 4.08 | 0.676 | 0.051 | 3.98 | 4.181 | 1.75 | 5 |
|  | Netherlands | 58 | 3.79 | 0.659 | 0.086 | 3.62 | 3.966 | 1 | 5 |
|  | United Kingdom | 105 | 3.76 | 0.804 | 0.078 | 3.605 | 3.916 | 1 | 5 |
|  | Total | 340 | 3.93 | 0.73 | 0.04 | 3.855 | 4.01 | 1 | 5 |
| **Self-Reported AI Literacy** | | | | | | | | | |
|  | Germany | 181 | 3.15 | 0.841 | 0.062 | 3.024 | 3.271 | 1 | 5 |
|  | Netherlands | 65 | 3.27 | 0.882 | 0.109 | 3.048 | 3.485 | 1.33 | 4.67 |
|  | United Kingdom | 109 | 3.2 | 0.876 | 0.084 | 3.036 | 3.368 | 1.33 | 5 |
|  | Total | 355 | 3.19 | 0.858 | 0.046 | 3.096 | 3.276 | 1 | 5 |
| **Support for Delegation of Decisions to Algorithms** | | | | | | | | | |
|  | Germany | 177 | 2.63 | 0.791 | 0.059 | 2.513 | 2.747 | 1 | 4.5 |
|  | Netherlands | 58 | 2.84 | 0.755 | 0.099 | 2.645 | 3.042 | 1.25 | 4.5 |
|  | United Kingdom | 105 | 2.56 | 0.785 | 0.077 | 2.408 | 2.711 | 1 | 5 |
|  | Total | 340 | 2.64 | 0.787 | 0.043 | 2.561 | 2.729 | 1 | 5 |
| **Transparency Expectations in Algorithmic Decision-Making** | | | | | | | | | |
|  | Germany | 176 | 4.5 | 0.498 | 0.038 | 4.424 | 4.572 | 3 | 5 |
|  | Netherlands | 58 | 4.21 | 0.649 | 0.085 | 4.036 | 4.378 | 2.25 | 5 |
|  | United Kingdom | 105 | 4.5 | 0.465 | 0.045 | 4.405 | 4.585 | 3.5 | 5 |
|  | Total | 339 | 4.45 | 0.527 | 0.029 | 4.391 | 4.504 | 2.25 | 5 |
| **Preference for Shared Decision-Making** | | | | | | | | | |
|  | Germany | 175 | 4.58 | 0.562 | 0.042 | 4.501 | 4.669 | 1.67 | 5 |
|  | Netherlands | 58 | 4.32 | 0.677 | 0.089 | 4.138 | 4.494 | 2.33 | 5 |
|  | United Kingdom | 105 | 4.48 | 0.615 | 0.06 | 4.364 | 4.602 | 2.67 | 5 |
|  | Total | 338 | 4.51 | 0.606 | 0.033 | 4.442 | 4.572 | 1.67 | 5 |
| **TRI Score** | | | | | | | | | |
|  | Germany | 210 | 3.19 | 0.564 | 0.039 | 3.113 | 3.267 | 1.5 | 4.56 |
|  | Netherlands | 86 | 3.38 | 0.517 | 0.056 | 3.273 | 3.494 | 2.06 | 4.75 |
|  | United Kingdom | 145 | 3.06 | 0.602 | 0.05 | 2.965 | 3.163 | 1.56 | 4.81 |
|  | Total | 441 | 3.19 | 0.578 | 0.028 | 3.132 | 3.24 | 1.5 | 4.81 |

**Table 22: Welch one-way ANOVA of country mean differences**

| Variable | Welch F | df1 | df2 | Raw p | FDR-adjusted p^a^ | η² |
| --- | --- | --- | --- | --- | --- | --- |
| Perceived Benefits and Support for Usage of PRP in MDM | 6.900 | 2 | 166.656 | .001 | .002 | .036 |
| Perceived Benefits and Support for Usage of AI in MDM | 7.843 | 2 | 148.122 | <.001 | .002 | .045 |
| Self-Reported AI Literacy | .478 | 2 | 161.621 | .621 | .621 | .003 |
| Delegation of Decisions to Algorithms | 2.646 | 2 | 150.811 | .074 | .086 | .015 |
| Transparency in Algorithmic Decision-Making | 5.168 | 2 | 138.582 | .007 | .012 | .043 |
| Shared Decision-Making | 3.972 | 2 | 139.708 | .021 | .029 | .026 |
| TRI Score | 9.145 | 2 | 227.258 | <.001 | .002 | .038 |

^a^For FDR correction, P values reported by SPSS as P<.001 were conservatively entered as P=.001.

Note: Welch one-way ANOVA was used because group sizes were unequal and homogeneity of variance could not be assumed for all scales. P values were adjusted across the seven exploratory country-level omnibus tests using the Benjamini-Hochberg false discovery rate procedure. Effect sizes for the statistically significant country differences were small, with η² ranging from .026 to .045. The combined “Other” category was excluded from inferential country comparisons because of its small and heterogeneous composition.

**Table 23: Games-Howell post-hoc comparisons of country mean differences**

| Scale | Comparison | Mean difference | p |
| --- | --- | --- | --- |
| Perceived Benefits and Support for Usage of PRP in MDM | Germany vs. Netherlands | 0.22 | .024 |
| Perceived Benefits and Support for Usage of PRP in MDM | Germany vs. UK | 0.23 | .003 |
| Perceived Benefits and Support for Usage of AI in MDM | Germany vs. Netherlands | 0.29 | .014 |
| Perceived Benefits and Support for Usage of AI in MDM | Germany vs. UK | 0.32 | .002 |
| Transparency in Algorithmic Decision-Making | Germany vs. Netherlands | 0.29 | .007 |
| Transparency in Algorithmic Decision-Making | UK vs. Netherlands | 0.29 | .010 |
| Shared Decision-Making | Germany vs. Netherlands | 0.26 | .021 |
| TRI Score | Netherlands vs. Germany | 0.19 | .013 |
| TRI Score | Netherlands vs. UK | 0.32 | <.001 |

Games-Howell post-hoc tests were used for pairwise comparisons following significant Welch tests. Only significant pairwise comparisons are shown. Mean differences are reported in the direction of the comparison shown.

**Table 24: Additional multiple linear regression associated with perceived support and benefits of PRP in MDM including TRI subdimensions (n=340)**

|  |  | |  |  | 95% CI | |
| --- | --- | --- | --- | --- | --- | --- |
| Coefficient | B | rSE^a^ | T | *P* | lower | upper |
| (constant) | .723 | .437 | 1.655 | .099 | -.136 | 1.582 |
| Self-Reported AI Literacy | .090 | .054 | 1.677 | .095 | -.016 | .195 |
| Delegation of Decisions to Algorithms | .249 | .048 | 5.243 | <.001 | .156 | .343 |
| Transparency in Algorithmic Decision-Making | .264 | .065 | 4.089 | <.001 | .137 | .391 |
| Shared Decision-Making | .119 | .064 | 1.845 | .066 | -.008 | .245 |
| Educational attainment | .038 | .026 | 1.485 | .138 | -.012 | .088 |
| Gender^b^ | .008 | .063 | .123 | .902 | -.117 | .133 |
| Age | .003 | .003 | 1.110 | .268 | -.002 | .009 |
| Optimism Score | .148 | .062 | 2.378 | .018 | .026 | .270 |
| Innovation Score | -.002 | .052 | -.031 | .975 | -.103 | .100 |
| Discomfort Score | -.019 | .045 | -.410 | .682 | -.108 | .071 |
| Insecurity Score | .017 | .049 | .345 | .730 | -.080 | .114 |

^a^ HC3 Method

^b^Gender coded as men=0 and women=1.

Note: This regression is reported to provide additional context and is not interpreted in the results section of the study. Discomfort and insecurity scores are not inverted, unlike the overall TRI score. The overall model was statistically significant, F(11,328)=14.34, P<.001, explaining 32.5% of the variance in perceived PRP benefits (R²=0.325; adjusted R²=0.302). The modified Breusch-Pagan test indicated significant heteroscedasticity, χ²(1)=7.51, P<.006. Therefore, HC3 robust standard errors were applied.

**Table 25: Additional multiple linear regression associated with perceived support and benefits of AI in MDM including TRI subdimensions (n=341)**

|  |  | |  |  | 95% CI | |
| --- | --- | --- | --- | --- | --- | --- |
| Coefficient | B | rSE^a^ | T | *P* | lower | upper |
| (constant) | 1.059 | .514 | 2.061 | .040 | .048 | 2.070 |
| Self-Reported AI Literacy | .261 | .064 | 4.056 | <.001 | .135 | .388 |
| Delegation of Decisions to Algorithms | .169 | .041 | 4.133 | <.001 | .089 | .250 |
| Transparency in Algorithmic Decision-Making | .197 | .064 | 3.096 | .002 | .072 | .321 |
| Shared Decision-Making | .110 | .065 | 1.684 | .093 | -.018 | .238 |
| Educational attainment | .035 | .026 | 1.334 | .183 | -.017 | .087 |
| Gender^b^ | -.208 | .078 | -2.669 | .008 | -.361 | -.055 |
| Age | .005 | .003 | 1.552 | .122 | -.001 | .011 |
| Optimism Score | .216 | .061 | 3.561 | <.001 | .097 | .336 |
| Innovation Score | -.143 | .055 | -2.607 | .010 | -.252 | -.035 |
| Discomfort Score | .004 | .049 | .074 | .941 | -.093 | .100 |
| Insecurity Score | -.135 | .051 | -2.672 | .008 | -.235 | -.036 |

^a^ HC3 Method

^b^Gender coded as men=0 and women=1.

Note: This regression is reported to provide additional context and is not interpreted in the results section of the study. Discomfort and insecurity scores are not inverted, unlike the overall TRI score. The overall model was statistically significant, F(11,329)=18.15, P<.001, explaining 37.8% of the variance in perceived AI benefits (R²=0.378; adjusted R²=0.357). The modified Breusch-Pagan test indicated significant heteroscedasticity, χ²(1)=17.26, P<.001. Therefore, HC3 robust standard errors were applied.
